# Genetic and Pathogenic Differentiation of *Fusarium oxysporum* Isolates from Ginger

**DOI:** 10.3390/jof12060390

**Published:** 2026-05-29

**Authors:** Andrea Matthews, Duy P. Le, Sharon Hamill, Jirah Villajuan, Donald M. Gardiner, Elizabeth A. B. Aitken, Andrew Chen

**Affiliations:** 1 School of Agriculture and Food Sustainability, The University of Queensland, St Lucia, QLD 4072, Australia; andrea.matthews@uq.net.au (A.M.); duy.le@dpird.nsw.gov.au (D.P.L.); jirahvilla1@gmail.com (J.V.); 2 New South Wales Department of Primary Industries and Regional Development, Narrabri, NSW 2390, Australia; 3 Maroochy Research Facility, Queensland Department of Primary Industries, Nambour, QLD 4560, Australia; sharon.hamill@dpi.qld.gov.au; 4 Queensland Alliance for Agriculture and Food Innovation, The University of Queensland, St Lucia, QLD 4072, Australia; donald.gardiner@uq.edu.au

**Keywords:** *Fusarium oxysporum* f. sp. *zingiberi*, ginger (*Zingiber officinale* Roscoe), Secreted In Xylem effectors, effector gene evolution, accessory sequences, plant–fungal interactions, endophytes, *Fusarium* yellows, comparative fungal genomics

## Abstract

Ginger (*Zingiber officinale* Roscoe) is a high-value horticultural crop widely cultivated for its culinary and medicinal applications, yet its production is increasingly constrained by soil-borne diseases. Among these, Fusarium yellows, caused by *Fusarium oxysporum* f. sp. *zingiberi* (*Foz*), is one of the most damaging constraints in ginger-growing regions around the world, leading to progressive yellowing, vascular blockage, and decline in rhizome quality. Members of the *Fusarium oxysporum* species complex are known to include both pathogenic and non-pathogenic lineages that often co-occur within the same host and environment, complicating disease diagnosis and epidemiological understanding. In this study, we examined *Fusarium*-like isolates recovered from both symptomatic and symptomless ginger plants within Southeast Queensland, the major ginger production region in Australia. We then investigated the genetic diversity, effector gene content, and pathogenic potential of these isolates. Comparative analyses revealed two genetically and functionally distinct groups: a clonal *Foz* lineage consistently associated with Fusarium yellows symptoms and characterised by a conserved set of Secreted In Xylem (SIX) effector genes (*SIX7*, *SIX9*, *SIX10*, and *SIX12*) and a diverse set of *F. oxysporum* isolates lacking these effectors. The conserved presence and co-localisation of *SIX7*, *SIX10*, and *SIX12* within a 5 kb region on a 1.4 Mb contig in the *Foz* lineage is consistent with the retention of a stable lineage-specific effector module, likely associated with accessory genomic regions that may contribute to host specificity and pathogenicity in the *Fusarium oxysporum* species complex. Pathogenicity assays confirmed that only the *Foz* lineage induced disease, whereas non-*Foz* isolates caused no visible symptoms despite limited colonisation of host tissues. These findings highlight the coexistence of pathogenic and endophytic *Fusarium* lineages within ginger production systems and support the use of effector-based markers for improved detection and disease management.

## 1. Introduction

Ginger (*Zingiber officinale* Roscoe) is a perennial, herbaceous plant of the Zingiberaceae family. It is believed to have originated in Southeast Asia, where the pungent, aromatic ginger rhizomes have been grown as an annual crop for thousands of years [1,2]. It is cultivated for domestic consumption in many cultures as a spice or vegetable and is also widely used in confectionery and beverages. Traditionally, ginger has also been used as a medicinal remedy for infections, nausea, inflammation, digestive and circulatory diseases [3,4]. It is grown commercially across tropical and subtropical regions, with a reported global production of over 4 million tonnes per annum [5].

In Australia, the most significant ginger rhizome diseases are caused by *Fusarium oxysporum* f. sp. *zingiberi* (*Foz*), *Pythium* species such as *P*. *myriotylum*, bacteria such as *Ralstonia solanacearum* or *R*. *sequeirae*, and root-knot nematodes (*Meloidogyne incognita* and *M*. *javanica*) [6,7,8,9]. Fusarium yellows, the name of the disease caused by *Foz*, is characterised by the appearance of bright yellow leaves among the dark green foliage of the ginger crop. Generally, the leaves at the base of the tiller turn yellow first, with the discolouration progressing up the tiller within a few days. The yellow leaves turn brown and papery, and newly emerging tillers may be stunted and discoloured. At harvest, the infected rhizomes will have extensive areas of dry, brown rot, rendering the rhizomes unmarketable [10]. The later disease stages are associated with the browning and rotting of the rhizomes, which is indicative of the colonisation and proliferation of the fungus. Once present in the soil, *Foz* can persist in the soil for a long time by surviving as resting spores in the absence of ginger hosts [11].

Fusarium yellows has been detected in many countries, including the United States [12], China [13] and Australia [9]. In Australia, commercial ginger production relies heavily on two cultivars (cv.), Canton and cv. Queensland, both of which have been found to be susceptible to *Foz* [9]. This lack of disease-resistant genotypes underscores a critical need for targeted research to understand the pathogenicity of *Foz* on ginger and, in turn, contribute towards better management of this disease. Therefore, a deeper understanding of the infection cycle with respect to host susceptibility and the diversity within the ginger-associated *Fusarium oxysporum* would be valuable to the Australian ginger industry.

Although *F. oxysporum* is widely known as a plant pathogen, many strains also exist as non-pathogenic endophytes that colonise plants without causing disease symptoms. These endophytic *F. oxysporum* strains have been reported in many plant species and can contribute to plant health by promoting growth and inducing resistance against pathogens [14,15,16]. Endophytic *F. oxysporum* strains often share close genetic relatedness with pathogenic forms and can colonise plant host tissues asymptomatically [17,18]. Some *F. oxysporum* endophytes may carry an effector gene repertoire, including the pathogenicity-associated Secreted In Xylem (SIX) genes [19]. While some of these effector genes have been implicated in disease symptom development and pathogenic interactions, the role of these effector genes during asymptomatic and endophytic interactions is yet to be understood. Furthermore, some endophytic strains can serve as biocontrol agents by directly or indirectly acting against pathogens through antagonistic mechanisms or competition for space and nutrients [20]. Therefore, they may represent a valuable resource for biocontrol strategies against *Foz* in managing Fusarium yellows.

The link between SIX effector gene profiles, pathogenicity, and genetic divergence in *F. oxysporum* associated with ginger remains unclear, limiting our understanding of pathogen emergence and the development of reliable diagnostics. In this study, we isolated a group of *F. oxysporum*-like isolates from ginger plants that were either healthy or showing Fusarium yellow symptoms. Screening using SIX effector gene markers showed the presence of four SIX genes consistently present in one group of isolates associated with pathogenicity on ginger. In contrast, non-pathogenic isolates lacked most of these SIX genes and caused no visible symptoms on ginger plants. These isolates were also genetically divergent from the *Foz* clade, suggesting that *Foz* in Australia likely arose from a single introduction event. The unique sequence variation observed in *Foz* SIX effector genes may provide a useful basis for developing diagnostic markers for detecting Fusarium yellows in the field.

Despite reports of *F. oxysporum* associated with ginger, the genetic basis of variation in pathogenicity remains unclear, particularly in relation to accessory genome structure and effector gene organisation. Comparative genomic analysis is therefore needed to resolve variation in these regions and to clarify how ginger-associated *F. oxysporum* isolates relate to diversity within the broader *Fusarium oxysporum* species complex. We addressed this by combining multilocus phylogenetic analysis with comparative genomics of isolates representing different host-specialisation lineages. In particular, we investigated the organisation and conservation of the *SIX7*–*SIX10*–*SIX12* effector region to assess differences in virulence-associated gene content between pathogenic lineages.

## 2. Materials and Methods

### 2.1. Fungal Isolates

Fifty-two *F. oxysporum* strains were isolated from ginger plants symptomatic or asymptomatic for Fusarium yellows in Southeast Queensland, Australia (Table S1). Monoconidial isolates were subsequently reisolated using hyphal tips of single germinated conidia.

### 2.2. Genes and PCR Primers

A set of universal primers for detecting *SIX1-14* genes was first used to detect SIX genes in these isolates [19]. Full-length *SIX7*, *SIX9*, *SIX10*, and *SIX12* gene sequences were retrieved from the existing *Foz* genome assemblies from NCBI under Bioproject PRJNA846078 [21] and then used to develop primers to amplify near-full-length copies of these genes from *Foz* (Table S2).

Translation elongation factor 1α (TEF-1α) and DNA-directed RNA polymerase II second largest subunit (RPB2) sequences from 150 *Fusarium* genome sequences were retrieved from NCBI and used as a backbone dataset for phylogenetic placement of our isolates. For SRA (NCBI, Sequence Read Archive) accessions where assemblies were not available, genome sequence data were assembled using SPAdes (v3.15.3) [22] prior to the sequence retrieval of these two genes. The backbone collection of *SIX7*, *SIX9*, *SIX10*, and *SIX12* genes was retrieved from the NCBI non-redundant database by performing tBLASTn (v 2.17.0+) searches using the amino acid sequences of these SIX genes from *Fusarium oxysporum* f. sp. *lycopersici* as query.

### 2.3. DNA Extraction and PCR

DNA was extracted from the mycelia of these isolates using a microwave method [23]. *TEF-1α* and *RPB2* genes were PCR-amplified using primers *EF1*/*EF2* [24] and *RPB2-5F2* [25]/*11aR* [26], respectively (Table S2). Some of the *RPB2* genes were difficult to amplify and instead were amplified as two overlapping fragments (860 bp + 917 bp) using primer pairs *5F2*/*7cR* and *7cF*/*11aR* (Table S2) [25,26]. A set of universal primers was used to detect the presence of *SIX1-14* genes in the *F. oxsporum* isolates obtained in this study [19]. *Foz*-specifc primers were then developed to amplify and retrieve full-length *SIX7*, *SIX9*, *SIX10* and *SIX12* genes using gene templates retrieved from *Foz* genome assemblies available in NCBI (VPRI4420, 44278, 44279, 44280) and *Foz* Pin2.2 long-read genome assembly (GCA_051624415.1). PCR products of the correct size were confirmed on a 1% agarose gel and then purified using a GeneJET PCR purification kit (Thermo Fisher Scientific, Waltham, MA, USA) and Sanger-sequenced at the Australian Genome Research Facility, Melbourne, Australia.

### 2.4. Phylogenetic Analysis

Geneious Prime v 2026.02 (Biomatter Pty Ltd., Auckland, New Zealand) was used for the phylogenetic reconstruction of the isolates. Firstly, multiple sequence alignment was performed using MAFFT v 7.490 [27]. The subsequent alignment was edited manually to minimise gaps. Multi-gene sequences were then concatenated and aligned to derive a consensus alignment sequence, which was then used as an input in MrBayes v 3.2.7 to construct phylogenetic trees using the Bayesian inference method [28]. Model selection was first performed in IQ-TREE v3 [29]. The best-fit models were identified under the corrected Akaike Information Criterion (AICc) or Bayesian Information Criterion (BIC) and were approximated using the closest available model specification, the general time-reversible model with invariant sites and gamma-distributed rate heterogeneity (GTR+I+G) in MrBayes. The running parameters used the GTR-I-G model of substitution with two independent analyses on four Markov chain Monte Carlo (MCMC) chains for 10,000,000 generations. Base frequencies were estimated using a uniform Dirichlet prior. A burn-in rate of 25% was selected to exclude all samples prior to the log-likelihood trace entering a stationary phase of the posterior distribution. Sampling was performed every 100 generations. *Fusarium verticillioides* (7600) was used as an outgroup to anchor the phylogenetic tree. The SIX gene phylogenies used 5,000,000 generations and were unrooted. Convergence was assessed using the average standard deviation of split frequencies.

Maximum Likelihood trees were inferred in IQ-TREE using 1000 ultrafast bootstrap replicates and 1000 SH-like approximate likelihood ratio tests (SH-aLRT). Nodes are annotated with ultrafast bootstrap percentages. The analysis was conducted under the best-fit model selected via ModelFinder.

The resulting consensus phylogenetic trees were visualised in iTOL (interactive Tree of Life, https://itol.embl.de, accessed on 26 May 2026) with either posterior probability (MrBayes) or bootstrap values (IQ-TREE) as branch support indicated at each node, and branch lengths scaled to represent the expected number of substitutions per site.

### 2.5. Plant Materials

Ginger plantlets of cultivar Canton were produced by in vitro tissue culture using the method and media as previously described [30] and then grown in a greenhouse to maintain a disease-free state at the Maroochy Research Facility, Queensland Department of Primary Industries, Queensland, Australia. Ginger cv. Canton rhizomes, approximately 40 g pieces, were planted into clean potting mix (Searles, Kilcoy, Australia) in 5 L poly bags (The Garden Superstore, Yandina, Australia). A total of 90 plants were arranged on two benches in a 50% shade house. The bags were initially watered by hand and then subsequently watered daily by an automatic overhead misting system. Plants were treated with a slow-release fertiliser (Osmocote Exact High K 8-9 month^®^) and subsequently with potassium sulphate at tiller emergence and thereafter as required.

### 2.6. Preparation of Inoculum

For inoculation, 15 isolates were chosen to represent *F. oxysporum* obtained from rhizomes grown in major ginger-producing regions of Southeast Queensland, and from fields with a range of crop histories prior to the ginger crop from which the rhizomes were sourced (Table S1).

The inoculum consisted of *F. oxysporum* spores extracted according to a previously published method [9,10]. To generate the spore suspensions, flasks of potato dextrose broth were inoculated with 4–5 mycelial plugs from plates colonised by each isolate and were then incubated for 7 days at 26 °C with shaking at 120 rpm on a rotating platform (Ratek, Victoria, Australia). Additional inoculum in the form of *F. oxysporum* infested Japanese millet seed (*Echinochloa esculenta* (A. Braun) H. Scholz) was prepared as previously described [31].

### 2.7. Plant Inoculation

On the day of inoculation, the bags containing the germinated ginger plants were arranged into 16 groups of five plants, so that the groups had an even mix of plant height and number of tillers per plant. Each plant had at least one semi-mature tiller (approx. 700 mm tall) and at least one smaller tiller. Bags were arranged onto two nursery benches on the same side of the greenhouse. Those designated for mock inoculation and those for endophyte inoculation were placed on one bench and those designated for *Foz* inoculation on the other bench. For each inoculation, approximately 15–20 g of colonised millet and 50 mL of fungal spore suspension were added to the soil. The inoculum was then lightly covered with more potting mix. Plants were maintained in the shade house for 8 weeks.

Plants were monitored for external symptoms of disease until harvest at 8 weeks post-inoculation. At harvest, external symptoms including the number of tillers, leaf discolouration and the height of the tallest tiller were recorded. Rhizomes were weighed and then split in half through the vertical axis. The cut surface was then rated on the percentage of the internal surface that was discoloured, as a measure of internal disease symptoms.

### 2.8. Reisolation of Fusarium oxysporum from Infected Plants

Four tissue pieces each from roots, rhizome, stem, and leaves were collected from two plants from each treatment. The stem samples comprised two pieces each taken at the rhizome shoulder and approximately 5 cm above the rhizome. The leaf pieces included the petiole and three pieces of the leaf blade transecting the central leaf rib. Tissue pieces were surface sterilised with 70% ethanol for a minute, air-dried, and plated onto half-strength potato dextrose agar (PDA) to detect the presence of colonies and spores typical of *F. oxysporum*.

A colony was considered to be *F. oxysporum*-like if it had abundant fluffy aerial and submerged mycelia, and the colony colour varied from white to pale violet to dark magenta on the media surface or underside of a PDA plate (Figure S1). Microconidia were produced in false heads on short monophialides and were generally abundant; oval, elliptical or reniform; single-celled; 3.0 to 5.0 µm wide and 6.0 to 14.0 µm long. Macroconidia produced in some isolates; typically, 3-septate; hyaline; slightly curved with an apical hook; approximately 3.0 to 5.0 µm wide and 20.0 to 30.0 µm long.

### 2.9. Statistical Analysis

Statistical analysis was conducted in Prism version 10.3.1. For rhizome discolouration, rhizome weight, and plant height, data were analysed using one-way ANOVA, followed by Tukey’s post hoc test, which was used to group means into homogeneous sets, with different letters above columns indicating significant differences at *p* ≤ 0.05. Plotted values represent means with 95% confidence intervals.

Using SPSS (IBM SPSS Statistics, v.29.0.1.0), a generalised linear model with a negative binomial distribution and log link was used to analyse the number of green tillers and yellow leaves at 8 weeks post-inoculation across all strains. Pairwise comparisons were performed using estimated marginal means with Bonferroni adjustment for multiple testing.

### 2.10. Genomics Analysis

#### 2.10.1. Genome Assemblies

To analyse the *F. oxysporum* genomic structure around the *SIX7-10-12* gene cluster, long-read genome assemblies were retrieved from the public NCBI genome database. These included *F. oxysporum* f. sp. *narcissi* (GCA_045837865.1), *F. oxysporum* f. sp. *lini* (GCA_013423245.1), *F. oxysporum* f. sp. *cepae* (GCA_003615085.1), *F. oxysporum* f. sp. *sesami* (GCA_017979615.1), *F. oxysporum* f. sp. *lycopersici* 4287 (GCA_000149955.2) and *F. oxysporum* f. sp. *zingiberi* (GCA_051624415.1). *F. oxysporum* f. sp. *cubense* assembly FocCAV2318 (Subtropical Race 4) was retrieved from Mycocosm (https://mycocosm.jgi.doe.gov/mycocosm/home). *Fusarium verticillioides* 7600 (GCA_000149555.1) was used as an outgroup to anchor the phylogenetic tree. All seven genomes were assessed for completeness using BUSCO v 5.8.0 [32] with the ascomycota_odb12 lineage dataset (v.2026-03-20-145944), and AUGUSTUS [33] with *Fusarium graminearum* as the species training model for gene prediction within the BUSCO pipeline.

#### 2.10.2. Structural Annotation of Protein-Coding Genes

Genome annotations were not available for the assemblies derived from *F. oxysporum* f. sp. *sesami*, *F. oxysporum* f. sp. *lini* and *F. oxysporum* f. sp. *narcissi*. For these three assemblies, repeat sequences in the genome were modelled using RepeatModeller v 2.0.4 [34] and then soft-masked using RepeatMasker v 4.1.5 [35]. Structural annotation of protein-coding genes was performed using BRAKER3 v3.0.6 [36]. Proteins from *F. oxysporum* f. sp. *lycopersici* 4287 were mapped to the soft-masked genomes, and the fungal model was applied during the GeneMark training step. AGAT v1.4.0 was used to identify and remove overlapping gene models and then retain only the longest isoform of each gene (https://zenodo.org/records/11106497, accessed on 26 May 2026).

#### 2.10.3. Collinearity Analysis

Collinearity at the chromosome level was investigated using the MCScanX v1.0.0 function of OrthoVenn3 [37] and six long-read genome assemblies of *F. oxysporum* f. sp. *lycopersici*, *F. oxysporum* f. sp. *zingiberi*, *F. oxysporum* f. sp. *narcissi*, *F. oxysporum* f. sp. *lini*, *F. oxysporum* f. sp. *cepae*, and *F. oxysporum* f. sp. *sesami*, while *F. oxysporum* f. sp. *cubense* CAV2318 was excluded due to its fragmented assembly. Genome-wide contig alignments were first generated using the NUCmer programme from the MUMmer package (v4.01) [38], with *F. oxysporum* f. sp. *lycopersici* used as the reference to determine chromosome number and order across the remaining five genomes. Alignments were then ordered according to the *F. oxysporum* f. sp. *lycopersici* reference using a custom script and visualised on the interactive dot plot tool online (https://dot.sandbox.bio, accessed on 26 May 2026) with default settings. For synteny analysis, core chromosomes with unique aligned regions across the five genomes were arranged according to the *F. oxysporum* f. sp. *lycopersici* reference, and the three to seven largest unaligned sequences per genome were also included. Two large non-aligned chromosomes, CM023981 and CM023982, from *F. oxysporum* f. sp. *lini* were excluded from this analysis. Corresponding GFF annotations were reordered, concatenated, and converted to BED format using a script provided by OrthoVenn3. Gene orthology clustering and collinearity analysis were performed in OrthoVenn3 using an e-value cutoff of 1 × 10^−15^ with all other parameters set to default. The resulting collinearity plot was interactively edited online, and the final SVG output was refined in Inkscape (v1.4.3).

#### 2.10.4. Manual Curation of SIX Gene Annotations

Genomes and their gene models were loaded into Geneious Prime v 2026.02 and local BLASTn and tBLASTn searches using a set of *SIX1-14* nucleotide or amino acid sequences were performed against all seven *F. oxysporum* assemblies to check their presence and absence in these genomes. For *SIX7*, *SIX10*, and *SIX12*, the coding domain sequence and their positions in each genome were carefully examined and then corrected using the *F. oxysporum* f. sp. *lycopersici* SIX gene models as a guide. SIX gene paralogues were assigned letters a, b, and c to differentiate their positions on the same or different chromosomes. The fixed gene annotations were exported from Geneious Prime in strict GFF3 format. All revised annotations were then processed with a custom script to correctly define gene–CDS relationships. The script parses each feature, assigns unique IDs to genes, mRNAs, exons, and CDSs, updates parent–child relationships, and ensures CDS phases are properly set. Features without a Name = tag are left unchanged, while annotations from other sources (e.g., GenBank, MAKER, Augustus) are preserved as provided. Gffread v 0.12.8 was then used to extract amino acid sequences from all seven genomes [39].

#### 2.10.5. Orthologue Clustering and Evolutionary Analysis

Gene Orthology and phylogenetic analysis of the annotated genomes were performed using OrthoVenn3 [40]. Gene-coding proteins from the seven genomes were clustered using OrthoFinder v3.0.1 [41] with an e-value threshold of 0.01 and an inflation value of 1.50. The evolution model was JTT-CAT. A divergence time of 5.34 million years ago (MYA) was estimated between *F. oxysporum* and *F. verticillioides*, using TimeTree5 [42]. Gene family expansion and contraction between genomes were calculated based on this calibrated evolutionary divergence time using CAFE5 [43].

#### 2.10.6. Orthologous Analysis Across the SIX7-10-12 Gene Cluster

The *SIX7*, *SIX10*, and *SIX12* reference genes from the *Foz* Pin2.2 reference genome were used as anchors to identify corresponding orthogroups across the other six *Fusarium* assemblies using the orthogroups output file from OrthoFinder. Using the orthogroup assignments, all orthologues, including any duplicated copies, were retrieved for each genome. Each gene was then linked back to its genome-specific GFF annotation to extract genomic coordinates, scaffold, and strand information using a custom R script. The final output was compiled into a structured ortholog table, listing the reference gene, its orthogroup, the genome in which it was found, gene ID, locus number, scaffold, start and end positions, and strand. This approach ensures that both single-copy and duplicated orthologues are captured, providing a complete mapping of SIX genes across all seven genomes. Visualisation of the genomic organisation of these orthologues was performed in R using the tidyverse, ggplot2, cowplot, and rtracklayer packages [44]. Gene coordinates were arranged along each scaffold, with large intergenic gaps compressed to highlight clustered regions while preserving their relative order. Genes are displayed as arrows oriented according to their direction of transcription. Boxes are added manually to indicate the start and end positions of each scaffold (genomic sequence, not drawn to scale) to provide genomic context.

## 3. Results

### 3.1. Detection of SIX Genes in Fusarium oxysporum Isolates from Ginger

Universal SIX gene profiles performed on 56 *Fusarium oxysporum* isolates showed that 38 isolates carried four SIX genes, namely *SIX7*, *SIX9*, *SIX10,* and *SIX12* (Table S3). These include five categorised strains that were previously isolated from ginger hosts symptomatic for Fusarium yellows (BRIP: 44963, 44967, 44969, 44971, 44977). Twelve endophytic isolates do not appear to carry any of these four SIX genes, whereas five of them (SP1.2, SP2, SP2.2, SP3.2, and SP4.2) only carry *SIX9* and not the other three SIX genes (Table S3).

### 3.2. Phylogenetic Placements of Fusarium oxysporum Isolates Within Fusarium oxysporum Species Complex

**Figure 1:**
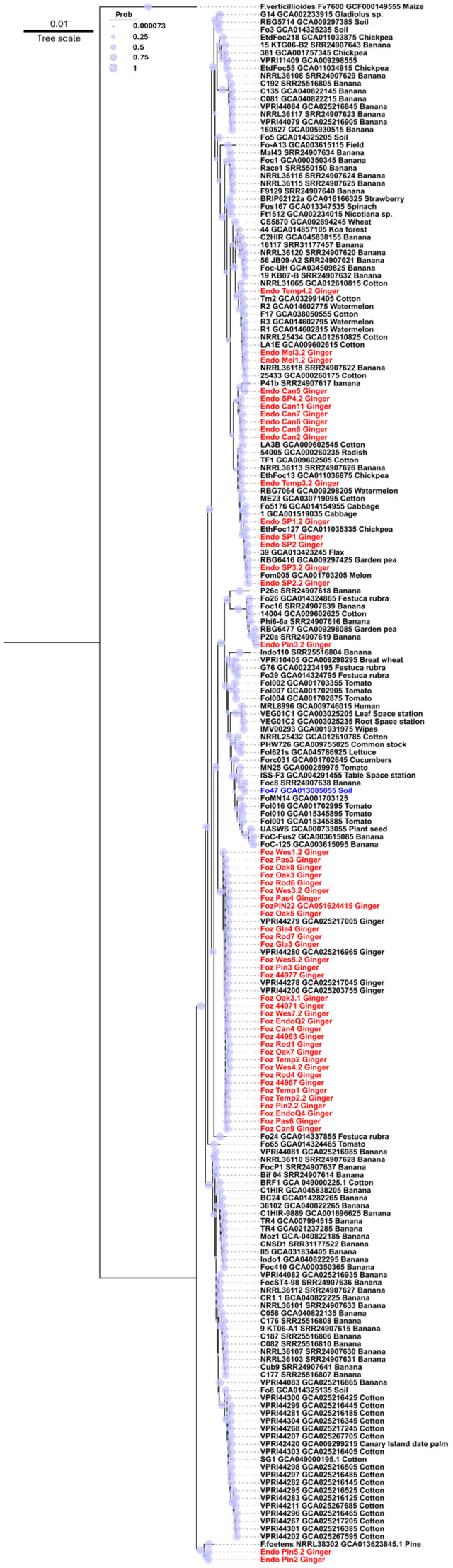
Bayesian phylogenetic tree reconstructed using concatenated sequences of translation elongation factor 1 alpha (*TEF-1α*) and the second largest subunit of RNA polymerase II (*RPB2*). The endophytic and pathogenic *Fusarium oxysporum* isolates from ginger analysed in this study are highlighted in red. The *F. oxysporum* strain Fo47 with known biocontrol properties is highlighted in blue. The bar denotes a scale range of 0.01. Circles indicate posterior probabilities expressed as a percentage at each node. *Fusarium verticillioides* (Fv7600) was used as an outgroup to anchor the phylogenetic tree.

Bayesian phylogenetic inference was performed in MrBayes using a concatenated alignment of *TEF1* and *RPB2* from approximately 200 taxa and 2.7 kb of sequence data per taxon. The convergence statistics showed an average standard deviation of split frequencies of 0.013 and an average potential scale reduction factor of 1.0. The analysis resolved a distinct *F. oxysporum* f. sp. *zingiberi* clade that includes 32 *Foz* isolates and the *Foz* reference strains (Figure 1). This phylogroup appeared to contain *Foz*-exclusive isolates. No genetic variation or single-nucleotide polymorphisms (SNPs) were detected in this phylogroup, suggesting that the *Foz* population belonged to a clonal lineage in Australia. A maximum likelihood analysis performed on this dataset produced a tree with the same topology (Figure S2).

A total of 17 *F. oxysporum* endophytes are phylogenetically positioned within a large clade, separated from the *Foz* clade (Figure 1). Within this group, several distinct subclades were observed. Mei1.2, Mei3.2 and Temp4.2 were clustered with a Race 1 strain of *F. oxysporum* f. sp. *cubense* (NRRL36118) [45], multiple races of *F. oxysporum* f. sp. *niveum* (Race 1–3) [46], *F. oxysporum* f. sp. *vasinfectum* strains F17 and 25433 (both Race 7) [47,48], as well as LA1E (MDS-12, race 4-like) [49]. This phylogroup also contained an *F. oxysporum* endophyte isolated from wheat (CS5870). The second subclade contained a large number of ginger endophytes, which include SP1, SP1.2, SP2, SP2.2, SP3.2, and Temp3.2. These isolates clustered together with multiple specialised forms, including *F. oxysporum* f. sp. *ciceris* (EthFoc127, EthFoc12), *F. oxysporum* f. sp. *lini* (isolate 39), *F. oxysporum* f. sp. *melonis* (Fom005), *F. oxysporum* f. sp. *niveum* (RBG7064), *F. oxysporum* f. sp. *pisi* (RBG6416), *F. oxysporum* f. sp. *cubense* (NRRL36113), and *F. oxysporum* f. sp. *raphanin* (54005). The third subclade contained Can2, Can5, Can6, Can7, Can11, and SP4.2. These isolates clustered with a *F. oxysporum* f. sp. *cubense* isolate P41b, previously characterised as a banana-infecting Race 1 strain [45].

A single ginger endophyte, Pin3-2, was clustered with a *F. oxysporum* f. sp. *cubense* strain P20a from banana and a *F. oxysporum* f. sp. *pisi* strain RBG6477. The banana-infecting strain was defined as a Race 1 strain and was isolated from the Silk subgroup banana cultivar ‘Mazano’ [45]. This cluster is nested within a broader clade that includes the known biocontrol agent Fo47, as well as specialised forms that infect banana, tomato, and other plant hosts (Figure 1). Interestingly, no other ginger endophytes were positioned within this clade. Ginger endophytes Pin2 and Pin5.2 clustered together with a *Fusarium foetens* strain NRRL 38302, confirming their *F. foetens* identity. This represents the first-ever report of *F. foetens* associated with ginger plants in Australia.

### 3.3. Sequence Analysis of the SIX Genes from the Foz Isolates

Full-length *SIX7*, *SIX9*, *SIX10*, and *SIX12* amino acid sequences from *F. oxysporum* f. sp. *lycopersici* were used to perform a tBLASTn search of the respective SIX gene homologs in the Pac-bio-derived assembly of *Foz* Pin2.2 (NCBI genome accession GCA_051624415.1). Single hits to a full-length copy of each SIX gene were identified in Pin2.2, suggesting that these four SIX genes are present as single-copy orthologues in the genome of *Foz* Pin2.2.

Full-length sequences of *SIX7*, *SIX9*, *SIX10*, and *SIX12* were obtained from 32 *Foz* isolates and were phylogenetically analysed using Bayesian inference and Maximum Likelihood methods, and a background dataset of the corresponding SIX gene sequences retrieved from 150 NCBI genome sequences (Figure 1). No SNPs were identified across all four SIX gene sequences among the 32 isolates, supporting the observation that they likely originate from a single lineage of *F. oxysporum* in Australia.

**Figure 2:**
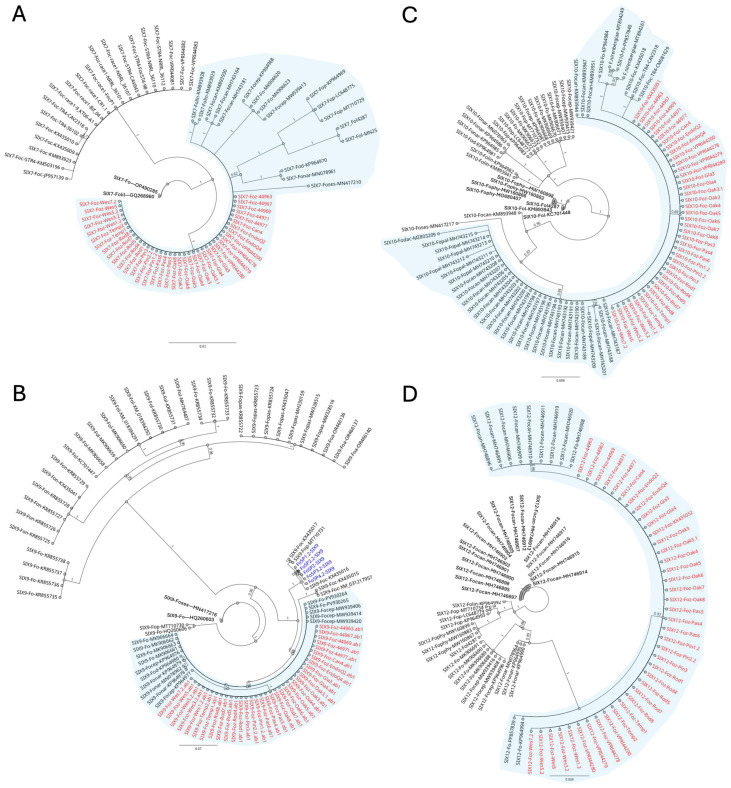
Bayesian phylogenetic analysis on the SIX genes present in the *Foz* isolates. Single SIX gene phylogenies derived from sequences within the *Fusarium oxysporum* species complex include (**A**) *SIX7*, (**B**) *SIX9*, (**C**) *SIX10*, and (**D**) *SIX12*. The endophytic and pathogenic *F. oxysporum* isolates from ginger analysed in this study are highlighted in blue and red, respectively. The scale bar indicates substitutions per site. Posterior probabilities are shown at each node. Trees are shown as unrooted. *Foz* phylogroups are shaded blue. Abbreviations included *F. oxysporum* f. sp. *cubense* (*Foc*), *F. oxysporum* f. sp. *niveum* (*Fon*), *F. oxysporum* f. sp. *sesami* (*Foses*), *F. oxysporum* f. sp. *cepae* (*Focep*), *F. oxysporum* f. sp. *Lycopersici* (*Fol*), *F. oxysporum* f. sp. *lilii* (*Folil*), *F. oxysporum* f. sp. *lini* (*Folin*), *F. oxysporum* f. sp. *canariensis* (*Focan*), *F. oxysporum* f. sp. *dianthi* (*Fod*), *F. oxysporum* f. sp. *narcissi* (*Fonar*), *F. oxysporum* f. sp. *pisi* (*Fop*), *F. oxysporum* f. sp. *palmarum* (*Fopal*), *Fusarium oxysporum* f. sp. *physali* (*Fophy*), and *F. oxysporum* f. sp. *dactylifera* (*Fodac*).

Phylogenetic analysis of *SIX7* revealed that *Foz* isolates clustered within a well-supported phylogroup comprising homologues from multiple *F. oxysporum formae speciales*, including *F. oxysporum* f. sp. *lini*, *F. oxysporum* f. sp. *canariensis*, *F. oxysporum* f. sp. *cepae*, *F. oxysporum* f. sp. *pisi*, *F. oxysporum* f. sp. *lycopersici*, *F. oxysporum* f. sp. *dianthi*, and *F. oxysporum* f. sp. *sesami* (Figure 2A). This phylogroup was distinct from a sister clade containing *F. oxysporum* f. sp. *cubense* Race 1, Subtropical Race 4, and Tropical Race 4 isolates. (Figure 2A, Figure S3).

*SIX9* sequences were resolved into several well-supported clades within *Fusarium oxysporum* species complex (Figure 2B, Figure S4). This indicates the presence of several distinct evolutionary lineages. *Foz SIX9* sequences formed a subgroup with homologues from *F. oxysporum* f. sp. *pisi*, *F. oxysporum* f. sp. *cepae*, *F. oxysporum* f. sp. *dianthi*, and *F. oxysporum* f. sp. *narcissi*, within a larger clade that also included *F. oxysporum* f. sp. *cubense*. A subset of ginger endophytes (SP1.2, SP2.2, SP2, SP3.2, and SP4.2) formed a divergent lineage within this clade. A separate lineage comprised homologues from *F. oxysporum* f. sp. *niveum*, *F. oxysporum* f. sp. *lycopersici*, *F. oxysporum* f. sp. *passiflorae*, and *F. oxysporum* f. sp. *albedinis*.

For *SIX10*, *Foz* sequences were grouped with a clade containing homologues from *F. oxysporum* f. sp. *canariensis*, *F. oxysporum* f. sp. *cubense* Tropical Race 4, as well as *F. oxysporum* f. sp. *dactylifera*, *F. oxysporum* f. sp. *palmarum*, and *Fusarium nirenbergiae* (Figure 2C, Figure S5). A second well-defined clade contained homologues from *F. oxysporum* f. sp. *lini*, *F. oxysporum* f. sp. *dianthi*, *F. oxysporum* f. sp. *narcissi*, *F. oxysporum* f. sp. *pisi*, and *F. oxysporum* f. sp. *cepae*, while sequences from *F. oxysporum* f. sp. *physali* and *F. oxysporum* f. sp. *lycopersici* were more distantly related.

*SIX12* formed a distinct clade comprising *Foz* sequences, within which homologues from *F. oxysporum* f. sp. *canariensis* grouped as a subclade (Figure 2D, Figure S6). In addition, a second, more divergent set of *SIX12* homologues from *F. oxysporum* f. sp. *canariensis* formed an independent phylogroup, suggesting the presence of duplicated *SIX12* copies in this *forma specialis*. To date, homologues belonging to this particular *SIX12* phylogroup have only been identified in *F. oxysporum* f. sp. *zingiberi* and *F. oxysporum* f. sp. *canariensis*.

Overall, the phylogenetic placement of *Foz* SIX genes indicates gene-specific evolutionary relationships within the *Fusarium oxysporum* species complex, with clustering patterns varying among genes and spanning multiple *formae speciales*. With the exception of *SIX9*, which is absent in *F. oxysporum* f. sp. *canariensis*, *Foz* shares closely related copies of the remaining three SIX genes (*SIX7*, *SIX10*, and *SIX12*) with this *forma specialis*.

### 3.4. Greenhouse Pathogenicity Testing Using Pathogenic and Endophytic Fusarium oxysporum Isolates

**Figure 3:**
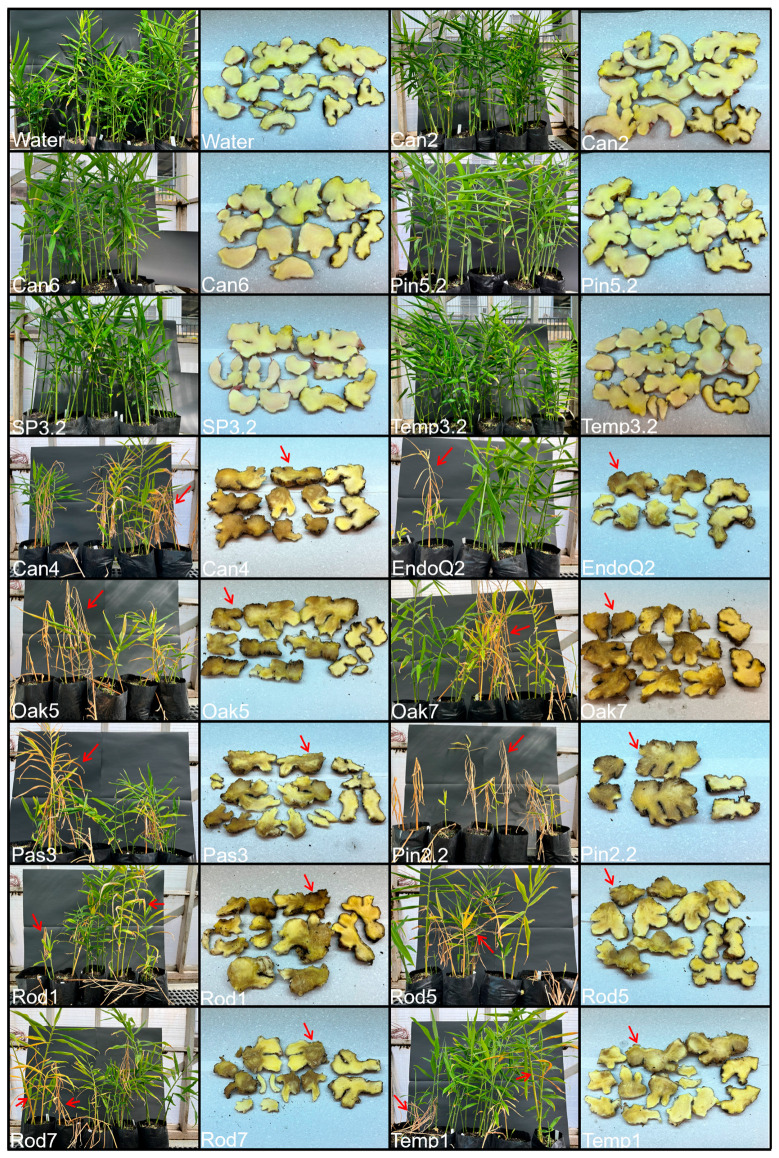
Symptomatology of ginger plants challenged with *Fusarium oxysporum* f. sp. *zingiberi* or endophytes at 8 weeks post-inoculation. Red arrows indicate yellow tillers on plants or necrotic lesions in the rhizomes of these plants.

Strains of these *F. oxysporum* isolates were further confirmed to be *Fusarium oxysporum* f. sp. *zingiberi* based on their ability to cause Fusarium yellows in ginger pot trials (Figure 3). *Foz* isolates Can4, EndoQ2, Oak5, Oak7, Pas3, Pin2.2, Rod1, Rod5, Rod7, and Temp1 all showed typical external symptoms of Fusarium yellows, including leaf and tiller yellowing (Figure 3). Disease severity appeared to vary amongst the treatments (isolates), with complete plant and tiller collapse observed in plants inoculated with Pin2.2 and Oak7. Disease expression was sporadic, with yellow tillers interspersed among healthy tillers within the same pot, which is a typical characteristic of Fusarium yellows (Figure 3). Internally, part or entire ginger rhizomes showed brown necrotic regions, indicative of the presence and proliferation of *Foz* in these issues.

Plants inoculated with the endophytic *F. oxysporum* strains (Can2, Can6, Pin5.2, Sp3.2, Temp3.2) remained healthy throughout the entire experiment. At harvest, these plants showed no visible external or internal symptoms of Fusarium yellows (Figure 3).

### 3.5. Plant Symptomatic Assessment and Comparisons Between Foz and Endophytes

**Figure 4:**
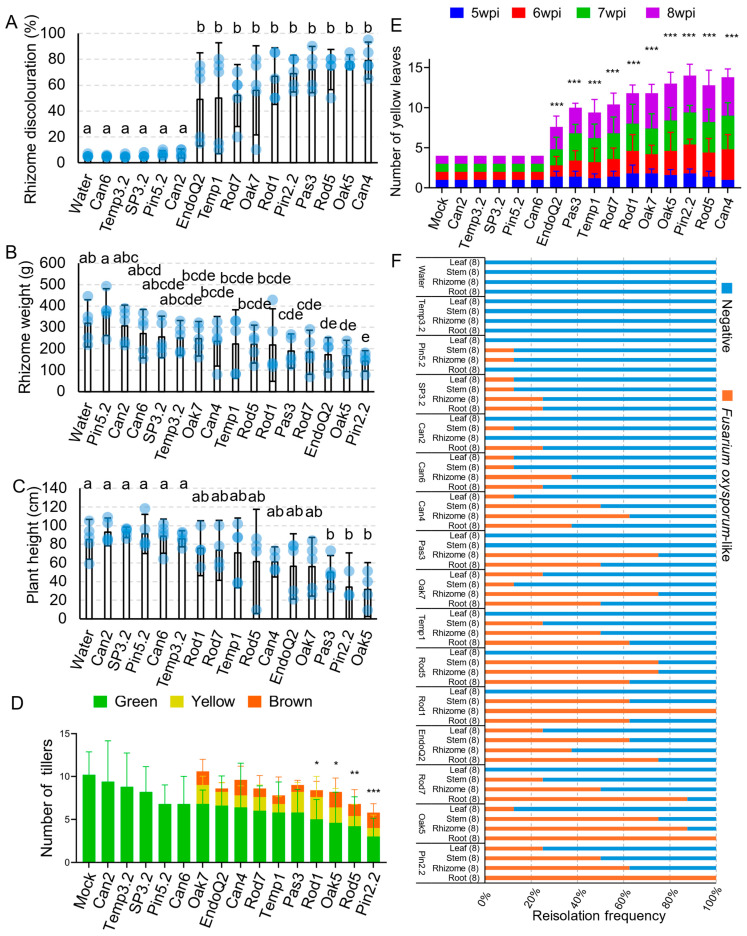
Plant pathogenicity pot trial using pathogenic and non-pathogenic *Fusarium oxysporum* isolates from ginger. Assessment of symptoms on plants inoculated with the five endophytes (Can2, Temp3.2, SP3.2, Pin5.2, and Can6), and the 10 *Foz* isolates. (**A**) Rhizome discolouration expressed as a percentage area of necrotic region over the entire rhizome. (**B**) Rhizome weight measured at harvest. (**C**) Plant height derived from the height of the tallest tiller. (**D**) The total number of tillers at harvest. (**E**) The number of yellow leaves at harvest. (**F**) Reisolation frequency of *F. oxysporum*-like colonies from the roots, rhizome, stems, and leaves of plants from each treatment group. Numbers in brackets indicate the number of tissues assessed for reisolation. (**A**–**C**) Matching superscript letters above data columns indicate that the group means are not significantly different from one another at *p* ≤ 0.05. Statistical significance relative to the water control for the number of green tillers (**D**) and yellow leaves (**E**) at 8 weeks post-inoculation is indicated as follows: * *p* ≤ 0.05, ** *p* ≤ 0.01, and *** *p* ≤ 0.001.

Rhizome discolouration clearly distinguished the two treatment groups, with *Foz* causing 50–80% discolouration in individual rhizomes, whereas no visible symptoms were observed in endophyte-inoculated plants (Figure 4A). The ranking of zhizome weight amongst the treatment groups is also consistent with their *Foz* susceptibility, with pathogenic forms clearly associated with reductions in weights and non-pathogenic forms maintaining rhizome weight relative to the untreated controls (Figure 4B). Although statistically significant, there is a large overlap in the separation of means as the dispersion of the dataset is large, which indicates considerable variability in the rhizome weight. Similarly, plant height shows that plants inoculated with the endophytes and the water control were significantly taller than those inoculated with *Foz* isolates Pas3, Oak5, and Pin2.2 at *p* = 0.05 (Figure 4C). The large overlap among mean plant height in the other *Foz*-inoculated plants reflects the sporadic occurrence of yellowed tillers among otherwise healthy tillers within individual pots.

Tiller number per treatment group indicates that tiller necrosis was associated with plant susceptibility to *Foz*, whereas endophyte-inoculated plants did not exhibit any yellowing symptoms (Figure 4D). Statistical modelling using a generalised linear model with a negative binomial distribution showed a significant overall strain effect on the number of green tillers observed at 8 weeks post-inoculation (Likelihood Ratio χ^2^ = 36.37, df = 15, *p* = 0.002). Several strains were significantly different from the water control, including Oak5 (*p* = 0.019), Rod1 (*p* = 0.040), Pin2.2 (*p* < 0.001), and Rod5 (*p* = 0.008).

The number of yellow leaves was counted weekly from 5 to 8 weeks post-inoculation. The results show that the number of yellow leaves remained consistently low at all time points in the plants inoculated with the five endophytes and the water control (Figure 4E). In contrast, the number of yellow leaves increased from week 5 onwards, and by week 8 was significantly higher than in endophyte-inoculated plants across all 10 *Foz*-inoculated groups at *p* = 0.05 (Figure 4E). The generalised linear model indicated a strong overall strain effect (Likelihood Ratio χ^2^ = 558.32, df = 15, *p* < 0.001). Pair-wise comparison against the water control indicated that all 10 *Foz* strains were significantly different (*p* < 0.001) when compared to the water control.

### 3.6. Reisolation of Fusarium oxysporum from Ginger Tissues at Harvest

Tissue reisolation from the leaf, stem, rhizome, and roots showed contrasting ability of pathogenic and non-pathogenic *F. oxysporum* to colonise ginger plants (Figure 4F). All tissues were negative for *F. oxysporum* in the water control and the endophyte Temp3.2-treated plants. Endophytes Pin5.2, SP3.2, Can2, and Can6 treated plants showed limited presence of *F. oxysporum* in some of the reisolated tissues (Figure 4F). The presence of *F. oxysorum* was detected in all 4 tissue types in SP3.2 and Can6 with an average range of 10 to 20% reisolation frequency. The *Foz* isolates were recovered the most frequently in the roots and rhizomes of ginger plants, with 100% reisolation frequencies detected in the roots of Pin2.2 and Oak5 inoculated plants, associating the presence of *Foz* with the rhizome discolouration observed in these plants (Figure 3 and Figure 4F). In the leaves, *Foz* was detected in 5 out of 10 *Foz*-inoculated treatment groups at approximately 20% reisolation frequency, suggesting that these isolates may have the ability to move up the aerial parts of the host plants. This is further supported by a high reisolation frequency of *F. oxysporum* detected in the stem of these plants. *Foz* was not detected in leaves of Pas3, Temp1, Rod5, Rod1, or Rod7 inoculated plants, although their presence was detected in the stems of four of these treated groups (Figure 4F). This suggests that the aggressiveness of *Foz* isolates may vary, and they may colonise the hosts and move up the aerial parts of the plants at different rates.

### 3.7. Comparative Genomics

#### 3.7.1. Rationale for Choosing Genomes for Comparative Analysis

Examination of *SIX7*, *SIX10*, and *SIX12* showed that all three genes are co-located within a 5182 bp region on contig 12 of the *Foz* Pin2.2 assembly. To investigate the structure of the virulence gene cluster containing these three SIX genes, and to explore the evolutionary relationships among *F. oxysporum* strains harbouring this cluster, we selected seven genome assemblies. These assemblies are highly contiguous, and each has a BUSCO completeness score exceeding 95% (Table S4). These *formae speciales* were initially selected based on the presence of these SIX genes shown in Figure 2. Genome assemblies were then selected based on the presence of this gene cluster on a single contig. Annotations of all *SIX7*, *SIX10*, and *SIX12* orthologues were manually inspected, corrected, and curated to account for any missing copies. The final curated protein-coding gene sets, excluding isoforms, were then used for the comparative analysis in Orthovenn3.

#### 3.7.2. Genome Structure Analysis

**Figure 5:**
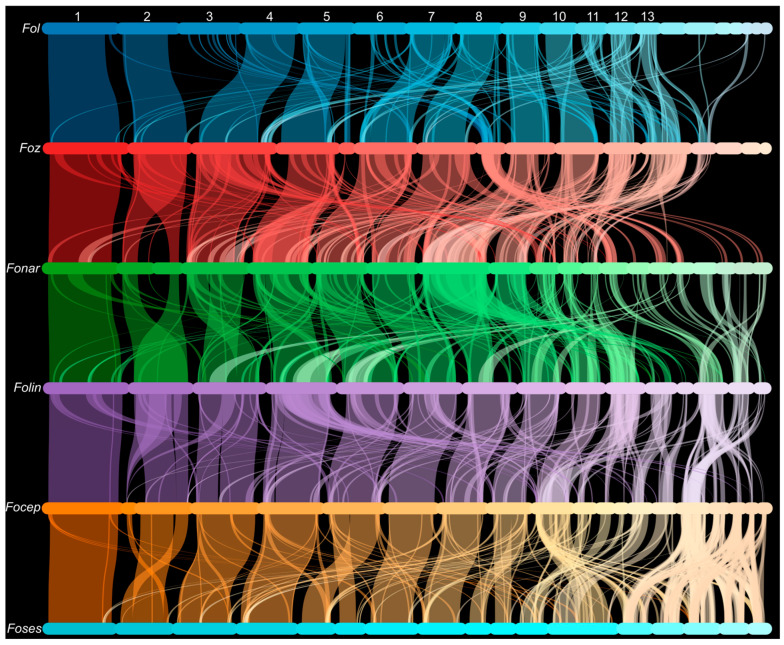
Chromosome synteny among genome assemblies of six *Fusarium oxysporum formae speciales*, including *F. oxysporum* f. sp. *lycopersici* (*Fol*), *F. oxysporum* f. sp. *zingiberi* (*Foz*), *F. oxysporum* f. sp. *narcissi* (*Fonar*), *F. oxysporum* f. sp. *lini* (*Folin*), *F. oxysporum* f. sp. *cepae* (*Focep*), and *F. oxysporum* f. sp. *sesami* (*Foses*). The 13 core chromosomes of *F. oxysporum* f. sp. *lycopersici* are shown as a reference. Conserved syntenic blocks are visualised across the chromosomes and/or contigs of each genome. Numbers corresponding to each *Fol* chromosome are indicated at the top of the diagram.

Collinearity analysis using MCScanX, as implemented in OrthoVenn3, revealed both conserved and lineage-specific chromosomal features across the six *Fusarium oxysporum formae speciales* (Figure 5). Notably, chromosomes 3 and 6 of *F. oxysporum* f. sp. *lycopersici* lacked syntenic counterparts in all five other genomes, indicating that they are lineage-specific chromosomes. In contrast, eight core chromosomes showed strong and consistent synteny across all six genomes, indicating a conserved core genome structure. Chromosomes 11–13 of *F. oxysporum* f. sp. *lycopersici* exhibited limited collinearity, with fragmented or partial alignments across multiple contigs of the other genomes, suggesting reduced conservation and possible structural rearrangements. Toward the right side of the plot, lineage-specific chromosomes 14 and 15 and other small scaffolds of *F. oxysporum* f. sp. *lycopersici* were not aligned to *F. oxysporum* f. sp. *zingiberi*, likely indicating divergent accessory genomic regions, as previously described for *F. oxysporum* f. sp. *lycopersici* [50]. In *F. oxysporum* f. sp. *zingiberi*, these small contigs also lacked detectable synteny with *F. oxysporum* f. sp. *narcissi*, whereas *F. oxysporum* f. sp. *narcissi* and *F. oxysporum* f. sp. *lini* displayed only sparse and discontinuous syntenic relationships in these regions. In contrast, *F. oxysporum* f. sp. *cepae* and *F. oxysporum* f. sp. *sesami* showed extensive multiple-to-multiple alignments among their unaligned contigs, indicative of highly repetitive or duplicated gene sequences shared between these genomes. Overall, syntenic relationships revealed variation in chromosomal conservation across the six *Fusarium oxysporum formae speciales*, with both conserved and lineage-specific genomic regions observed.

#### 3.7.3. Orthologous Clustering Analysis

**Figure 6:**
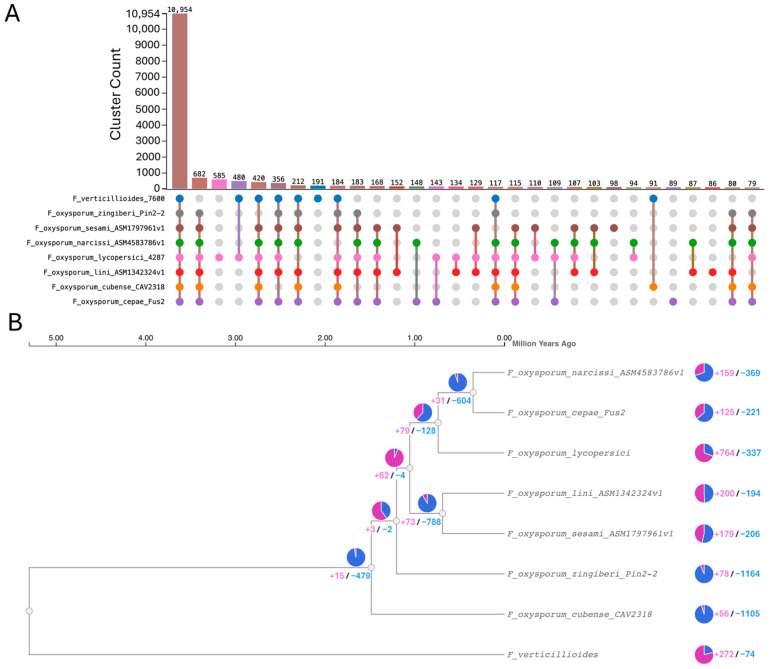
Comparative genomic analysis of *Fusarium oxysporum* f. sp. *zingiberi* genome Pin2.2 and other *F. oxysporum* pathogen genomes carrying some or all of the *SIX7*, *SIX10*, and *SIX12* genes. (**A**) Upset plot of orthologous clusters identified using OrthoVenn3, highlighting the top 30 conserved orthogroup clusters across genomes and genome pairwise comparisons. (**B**) Time-calibrated phylogenetic tree generated using CAFE5, showing evolutionary relationships among *F. oxysporum* genomes and associated gene family gains (purple) and losses (blue) over time. The timeline is calibrated using an estimated divergence of 5.3 MYA between *F. verticillioides* and *F. oxysporum*.

Protein clustering analysis using Orthovenn3 identified 20,094 conserved orthologous clusters across *Fusarium oxysporum* f. sp. *zingiberi* (Pin2-2, GCA_051624415.1), *F. oxysporum* f. sp. *lycopersici* (Fol4287, GCA_000149955.2), *F. oxysporum* f. sp. *cubense* (CAV2318_1), *F. oxysporum* f. sp. *narcissi* (AJ275, GCA_045837865.1), *F. oxysporum* f. sp. *lini* (strain #39, GCA_013423245.1), *F. oxysporum* f. sp. *cepae* (FoC_Fus2, GCA_003615085.1), and *F. oxysporum* f. sp. *sesami* (MR4003, GCA_017979615.1). *Fusarium verticillioides* (7600, GCA_000149555.1) was included as the outgroup. A core set of 10,954 orthologous genes was identified (Figure 6A), of which 6807 were single-copy across all eight genomes (Table S5). The seven *F. oxysporum* strains shared 682 orthologues exclusively.

Unique gene clusters were most abundant in *F. oxysporum* f. sp. *lycopersici* (582 clusters/1862 genes), followed by *F. oxysporum* f. sp. *sesami* (98/261), *F. oxysporum* f. sp. *cepae* (89/273), *F. oxysporum* f. sp. *lini* (86/220), *F. oxysporum* f. sp. *narcissi* (50/129), *F. oxysporum* f. sp. *zingiberi* (12/29), and *F. oxysporum* f. sp. *cubense* (8/20). GO term enrichment analysis indicated that *F. oxysporum* f. sp. *zingiberi* is enriched for oxidoreductase activity (3 genes, *p* = 0.0008). The top two most significant GO terms for the other *formae speciales* were: pathogenesis (2 genes, *p* = 4.66 × 10^−19^) and metal ion binding (2 genes, *p* = 1.05 × 10^−11^) for *F. oxysporum* f. sp. *lini*; regulation of transcription (2 genes, *p* = 1.51 × 10^−6^) and mycotoxin catabolic process (4 genes, *p* = 0.0001) for *F. oxysporum* f. sp. *narcissi*; oxidoreductase activity (7 genes, *p* = 1.17 × 10^−11^) and metal ion binding (6 genes, *p* = 1.27 × 10^−11^) for *F. oxysporum* f. sp. *cepae*; and oxidoreductase activity (4 genes, *p* = 3.05 × 10^−104^) and zinc ion binding (2 genes, *p* = 4 × 10^−19^) for *F. oxysporum* f. sp. *sesami*. No significant GO enrichment was detected for *F. oxysporum* f. sp. *cubense*. Overall, oxidoreductase activity and metal ion binding are recurrently enriched among the unique gene clusters across multiple *F. oxysporum formae speciales*.

#### 3.7.4. Gene Family Expansion and Contraction Analysis

Gene family evolution analysis using CAFE5 revealed distinct patterns of gene family expansion and contraction over an estimated 5.3 million years since the divergence of *Fusarium oxysporum* and *Fusarium verticillioides* (Figure 6B). Within *F. oxysporum*, an early divergence occurred at ~1.5 MYA, separating *F. oxysporum* f. sp. *cubense* (CAV2318) from the remaining lineages. A subsequent split at ~1.2 MYA gave rise to *F. oxysporum* f. sp. *zingiberi* (Pin2.2) and the lineage leading to the remaining *formae speciales*. These early-diverging lineages are characterised by pronounced gene family contraction, with 1105 and 1164 contracted families observed in *F. oxysporum* f. sp. *zingiberi* and *F. oxysporum* f. sp. *cubense*, respectively, alongside relatively limited expansion (56 and 78 gene families, respectively). The outgroup, *F. verticillioides*, exhibited moderate gene family dynamics, with 272 expansions and 74 contractions.

These patterns highlight the early divergence of the *F. oxysporum* f. sp. *cubense* strain CAV2318 from *F. oxysporum* f. sp. *zingiberi* isolate Pin2.2, with both lineages being distinct from the clade giving rise to the remaining *formae speciales*. Within this latter lineage, two well-supported subgroups are apparent. The first comprises *F. oxysporum* f. sp. *lini* and *F. oxysporum* f. sp. *sesami*, both of which display relatively balanced gene family dynamics (*F. oxysporum* f. sp. *lini*: 200 expansions/198 contractions; *F. oxysporum* f. sp. *sesami*: 179 expansions/206 contractions). The second subgroup includes *F. oxysporum* f. sp. *lycopersici*, which shows substantial gene family expansion (764 gained vs. 337 lost), alongside the closely related *F. oxysporum* f. sp. *cepae* and *F. oxysporum* f. sp. *narcissi*, both of which exhibit a net loss of gene families (*F. oxysporum* f. sp. *cepae*: +125 expansions/−221 contractions; *F. oxysporum* f. sp. *narcissi*: +159 expansions/−369 contractions).

Overall, with the exception of *F. oxysporum* f. sp. *lycopersici* and *F. oxysporum* f. sp. *lini*, most *formae speciales* display a general trend toward gene family contraction, with the most extensive losses observed in the early-diverging *F. oxysporum* f. sp. *cubense* and *F. oxysporum* f. sp. *zingiberi* lineages.

#### 3.7.5. Analysis of the *SIX7*, *SIX10* and *SIX12* Virulence Gene Cluster

**Figure 7:**
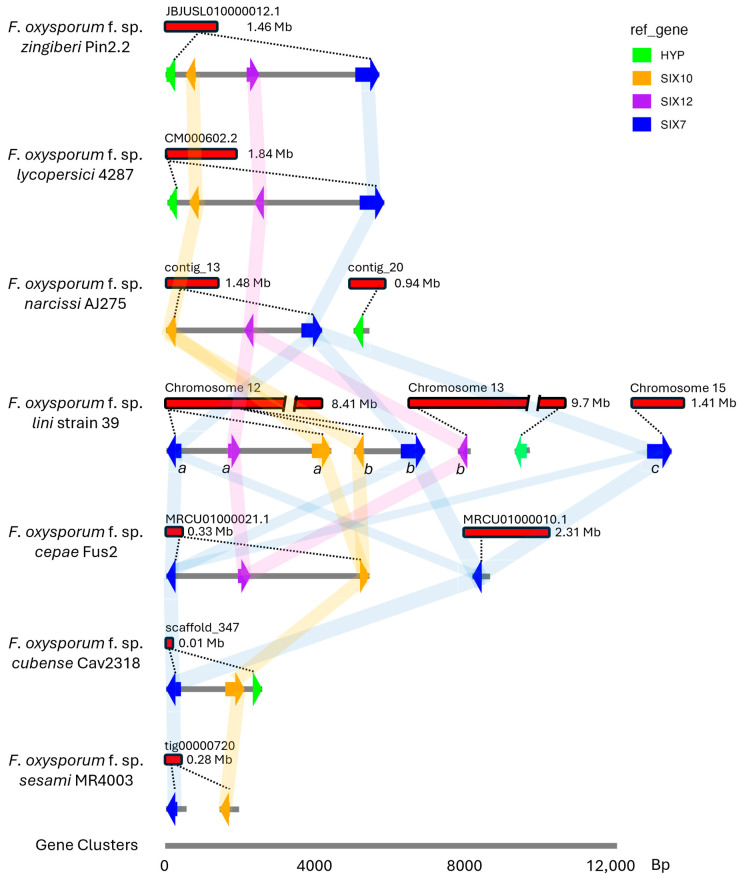
Gene collinearity across the *SIX7*, *SIX10*, and *SIX12* target region was evaluated by comparing gene content, order, and orientation within this genomic region across seven *Fusarium oxysporum* genome assemblies. Gene clusters are shown to scale according to the grey scale bar at the bottom. Contigs (red bars) are not drawn to scale, but their lengths are indicated at the end of each bar. Dashed lines indicate the positions of specific gene clusters on the contigs. Arrows represent the direction of transcription. Genes are colour-coded as *SIX7* (blue), *SIX10* (orange), *SIX12* (purple), and *HYP* (hypothetical proteins, green). Light-coloured links indicate gene orthology across genomes as determined by OrthoFinder v3.

Comparative analysis of gene collinearity across the *SIX7*, *SIX10*, and *SIX12* regions revealed both conservation and structural variation among *F. oxysporum* genomes (Figure 7). *F. oxysporum* f. sp. *zingiberi* (Pin2.2) contains a complete *SIX7*-*SIX10*-*SIX12* gene cluster. *F. oxysporum* f. sp. *cubense* Race 1 strain Cav2318, an earlier diverging lineage, only carries *SIX7* and *SIX10* together.

Genomes within this lineage can be divided into two distinct subclades (Figure 6). The first subclade, including *F. oxysporum* f. sp. *lycopersici* (4287), *F. oxysporum* f. sp. *narcissi* (AJ275) and *F. oxysporum* f. sp. *cepae* (Fus2), shows strong conservation of the entire gene cluster, with maintained gene content, order, and orientation (Figure 7).

In contrast, the second subclade, represented by *F. oxysporum* f. sp. *lini* (strain 39) and *F. oxysporum* f. sp. *sesami* (MR4003), exhibits substantial structural variation (Figure 7). In *F. oxysporum* f. sp. *lini* strain 39, the cluster is duplicated and redistributed with one complete cluster retained, while a partial duplicate containing *SIX7* and *SIX10* is translocated within chromosome 12. Additional copies of *SIX7* and *SIX12* are located on different chromosomes, resulting in three copies of *SIX7* and two copies of each *SIX10* and *SIX12* (Figure 7). Conversely, *F. oxysporum* f. sp. *sesami* MR4003 contains only *SIX7* and *SIX10* on a ~0.28 Mb contig, with no detectable *SIX12*. These genes are not arranged as a contiguous cluster, indicating a reduced and structurally fragmented gene complement.

Across all genomes analysed, SIX genes are frequently located near contig ends, suggesting an enrichment in telomeric or subtelomeric regions.

## 4. Discussion

Secreted In Xylem effectors are small, secreted proteins delivered into the plant vasculature during infection and are widely implicated in the pathogenicity of *F. oxysporum* across diverse host species [51,52,53]. Screening for SIX genes in this study revealed that all pathogenic *Foz* isolates consistently carried the same combination of effectors, *SIX7*, *SIX9*, *SIX10*, and *SIX12*, whereas non-pathogenic isolates lacked most SIX genes, with some carrying *SIX9*. This clear partitioning between pathogenic and non-pathogenic isolates aligns with previous studies demonstrating that SIX effector repertoires are strongly associated with host specificity and virulence in *F. oxysporum* [54,55,56].

### 4.1. Clonal Nature of the Australian Foz Population

Phylogenetic analysis based on the concatenated sequences of *TEF1* and *RPB2* genes grouped all *F. oxysporum* f. sp. *zingiberi* isolates collected in this study into a single, well-supported clade. This clade also included previously characterised reference *Foz* strains, providing strong molecular evidence that the isolates examined here belong to the same *forma specialis*. The clustering of all pathogenic isolates together, with the absence of sequence variation across the concatenated loci among the 35 *Foz* isolates, indicates a high degree of genetic uniformity and supports the inference that the Australian *Foz* population is largely clonal, consistent with earlier reports on the genetic homogeneity and vegetative compatibility among *Foz* isolates in ginger-growing regions in Australia [6,9,57]. Similar clonal structure is observed in other *F. oxysporum* lineages, such as the banana-infecting Tropical Race 4 strains, which also exhibit very low genetic diversity (Figure 1) [58]. A clonal pathogen population can spread rapidly, especially in a susceptible clonal host crop, as is the case for banana and ginger cultivars in Australia. The conserved presence of these four SIX genes across pathogenic *Foz* isolates highlights their potential to be leveraged as robust molecular markers for identifying pathogenic strains from ginger.

### 4.2. In-Field Diagnostics for the Australian Foz Population

A practical application of these effector-based markers is in the development of rapid field diagnostics such as loop-mediated isothermal amplification (LAMP) assays. LAMP can be designed to target specific effector genes that are consistently present in the pathogenic *Foz* isolates, allowing quick and sensitive detection directly from plant or soil samples. Similar approaches have already been developed for the detection of *F. oxysporum* f. sp. *cubense* Tropical Race 4. Ordóñez et al. developed a LAMP assay using unique genomic markers for rapid in planta detection of Tropical Race 4 [59], while Arrieta Salgado et al. used comparative genomics to develop a portable in-field LAMP diagnostic assay [60]. These studies demonstrate the potential for translating stable pathogen-specific genomic signatures into practical tools for rapid detection and disease management.

### 4.3. Endophytic Strains That Colonise Ginger Hosts Asymptomatically

A diverse set of *F. oxysporum* isolates recovered from asymptomatic ginger plants clustered across multiple lineages within the *F. oxysporum* species complex (Figure 1). These isolates were phylogenetically associated with several known *formae speciales*, including *F. oxysporum* f. sp. *cubense*, *F. oxysporum* f. sp. *vasinfectatum*, *F. oxysporum* f. sp. *niveum*, *F. oxysporum* f. sp. *cepae*, *F. oxysporum* f. sp. *pisi*, and *F. oxysporum* f. sp. *lycopersici*. Despite their placement within or adjacent to pathogenic lineages of other hosts, these isolates did not induce Fusarium yellows symptoms in ginger under glasshouse conditions. Notably, the majority of these isolates also lacked the core SIX effector genes detected in pathogenic *Foz* isolates, further distinguishing them from the pathogenic lineage identified in this study. Together, these patterns are consistent with the well-documented ecological continuum within *F. oxysporum* and related plant-associated fungi, where isolates can exist as pathogens or endophyte-like forms depending on host association and environmental context [61,62,63]. Non-pathogenic *F. oxysporum* strains have also been reported to occur alongside pathogenic lineages in soil and plant-associated environments, without causing disease in non-host plants [64,65]. Accordingly, these isolates may represent either non-pathogenic lineages or members of host-specific *formae speciales* associated with other plant hosts that are unable to infect ginger and may act as asymptomatic or transient colonisers within ginger-associated environments. However, their precise ecological status cannot be definitively resolved based on phylogenetic placement and SIX gene content alone.

### 4.4. Role of SIX Gene Effectors in Virulence and Host Specificity

SIX gene effectors are associated with pathogenicity in *Fusarium oxysporum* and may play roles in host–pathogen interactions during infection. While the exact functions of all SIX proteins are not fully understood, studies in tomato show that some effectors suppress plant immune responses and promote colonisation [56,66]. Different SIX genes can also interact with specific plant resistance proteins, which can determine whether a plant variety is resistant to particular *F. oxysporum* strains [55,67].

Our phylogenetic trees showed that *Foz* SIX genes formed subgroups with the corresponding SIX effectors from specialised forms of *F. oxysporum* on other hosts. This indicates that even distantly related *formae speciales* may share similar effectors due to evolutionary constraints [67] or horizontal transfer [68]. These shared effectors may maintain structural functions needed for infection even if their sequences have evolved and diverged [69]. In *F. oxysporum* f. sp. *lycopersici*, chromosomal deletions encompassing the *SIX7*–*SIX10*–*SIX12* cluster did not result in reduced pathogenicity on tomato, indicating that these genes are dispensable for *Fol* virulence on its host [70]. However, comparative genomic analyses have revealed highly conserved homologues of this cluster in other *formae speciales*, including *F. oxysporum* f. sp. *physali*, leading to the hypothesis that this genomic region may be undergoing adaptive diversification in response to alternative host environments [71]. Supporting this notion, recent functional evidence implicates *SIX10* in pathogenicity in other host systems, such as strawberry, suggesting that its contribution to virulence may be context-dependent [72]. Together, the presence of *SIX7*, *SIX10*, and *SIX12* in *Foz* may reflect a conserved but functionally flexible effector repertoire, potentially involved in host-specific interactions rather than core pathogenicity.

### 4.5. The Comparative Analysis of the SIX7-SIX10-SIX12 Gene Cluster in Fusarium oxysporum

Overall, genome synteny analysis highlights patterns consistent with a conserved core genome structure alongside dynamic accessory regions contributing to genomic diversity and evolution within *F. oxysporum* species complex [50]. The patterns of conservation and structural variation observed across the *SIX7*–*SIX10*–*SIX12* region point to a highly dynamic evolutionary landscape within *F. oxysporum*. Only two *F. oxysporum* f. sp. *cubense* genomes (Cav2318 Race 1 and 36102 Tropical Race 4) have been reported to contain both *SIX7* and *SIX10* together, and none include the full cluster with *SIX12* [73]. The presence of a complete gene cluster in *F. oxysporum* f. sp. *zingiberi* (Pin2.2), but not in *F. oxysporum* f. sp. *cubense* Cav2318, suggests that *SIX12* was likely acquired after divergence from a lineage containing only *SIX7* and *SIX10*, consistent with the modular evolution of effector repertoires in this species complex [50,74].

The strong conservation of the full cluster in *F. oxysporum* f. sp. *lycopersici*, *F. oxysporum* f. sp. *narcissi*, and *F. oxysporum* f. sp. *cepae*, despite differences in plant host range (dicots versus monocots), suggests that this gene set is maintained across divergent lineages, potentially reflecting a role in host–pathogen interactions rather than a universally required virulence function. This aligns with previous work showing that SIX genes can be maintained across lineages where they contribute to host colonisation and pathogenic fitness [56,75].

Among the closest related *F. oxysporum* f. sp. *lini* strain 39 and *F. oxysporum* f. sp. *sesami* MR4003 genomes, variation is observed in the *SIX7*–*SIX10*–*SIX12* genomic region, indicating recent diversification of this accessory gene cluster. In *F. oxysporum* f. sp. *lini* strain 39, duplication and redistribution of these genes across multiple chromosomes are consistent with segmental duplication and rearrangement events. Segmental duplications have been shown to contribute to the expansion and structural evolution of accessory genomic regions in *F. oxysporum* [45]. These duplications may contribute to the expansion of effector-rich regions and the formation of novel gene combinations for virulence and host range. Conversely, the smaller gene complement observed in *F. oxysporum* f. sp. *sesami* MR4003, including the absence of *SIX12*, suggests an evolutionary trajectory characterised by gene loss within this cluster. In contrast to the expansion observed in *F. oxysporum* f. sp. *lini*, these differences suggest that closely related lineages can show divergent patterns of virulence gene content, potentially reflecting different selective pressures imposed by their respective hosts [76].

Finally, the localisation of SIX genes near telomeric or subtelomeric regions across genomes further supports their association with accessory genomic compartments. These regions are generally dynamic and enriched for structural variation, and are thought to facilitate effector evolution through recombination, duplication, and rearrangement [77,78]. Notably, recent work has shown that accessory regions can expand through repeated segmental duplications, highlighting their contribution to the evolution of genomic diversity in pathogenic *F. oxysporum* lineages [45].

### 4.6. Fusarium Foetens Associated with Ginger Hosts

The detection of *F. foetens* in the ginger in this study is unexpected as *F. foetens* has not previously been detected in ginger plants. *F. foetens* was originally described by Schroers et al. (2004), following its isolation from *Begonia* × *hiemalis* hybrids (*Elatior begonias*) showing symptoms of basal rot, vein yellowing, and wilting in the Netherlands [79]. It was subsequently reported as a pathogen of begonia in other European countries [80], as well as in the United States [81] and Canada [82]. *F. foetens* can be distinguished genetically from members of its sister taxa, *Fusarium oxysporum*, by sequencing and concatenation of the *TEF1*-α and *RPB2* genes (Figure 1) or *TEF1-α*, *β-tubulin*, and *mtSSU* rRNA genes [79]. Morphologically, *F. foetens* is characterised by occasional production of polyphialides, relatively long monophialides intermingled with shorter monophialides, distinct sporodochial conidiomata and a characteristic colony odour [79].

Since its initial taxonomic description, *F. foetens* has subsequently been detected in South Africa as a pathogen of rooibos [83] and in China as a pathogen of potato [84], tobacco [85], lavender [86] and sweet potato [87]. It has also been detected in cassava in Brazil [88]. *F. foetens* was first detected in Australia in undisturbed soil [89,90], approximately 3000 km from the current detection in Queensland. While *F. foetens* was found not to cause symptoms in ginger, its presence in Queensland indicates that, as a species, it is likely to have a much more widespread distribution and possibly a wide pathogenic or endophytic potential.

## 5. Conclusions

In conclusion, this study demonstrates a clear genetic and functional separation between pathogenic *F. oxysporum* f. sp. *zingiberi* and asymptomatic endophytic *F. oxysporum* isolates associated with ginger in Australia. The pathogenic *Foz* population was highly clonal and consistently characterised by a conserved set of effector genes comprising *SIX7*, *SIX9*, *SIX10*, and *SIX12*. In comparison, endophytic isolates lacked these core effectors, were phylogenetically distinct, and showed contrasting host–microbe interactions. Glasshouse pathogenicity assays confirmed that only *Foz* isolates induced Fusarium yellows, leading to severe rhizome necrosis, reduced plant vigour, and successful tissue colonisation, while plants inoculated with endophytes remained asymptomatic or showed only weak colonisation. Comparative genomic analyses further revealed that the *SIX7*–*SIX10*–*SIX12* cluster showed lineage-specific patterns of conservation, duplication, loss, and rearrangement across these *F. oxysporum* strains, consistent with modular evolution of effector genes. Together, these findings support the observation that *Foz* represents a recently introduced, genetically uniform pathogen in Australia with a stable effector signature that can be exploited for diagnostics, while endophytic *F. oxysporum* represents a diverse and ecologically distinct component of the ginger microbiome with potential biocontrol relevance.

## Data Availability

All analysis outputs generated in this study are provided within the article and its Supplementary Materials. The Pin2.2 genome assembly and its annotation files are available at NCBI, under genome accession GCA_051624415.1. The raw Pac-bio and Illumina data are publicly available within the sequence read archive SRR31741969, under Bioproject PRJNA1195658. *TEF-1α*, *RPB2*, *SIX7*, *SIX9*, *SIX10*, and *SIX12* gene sequences used for creating the phylogenetic trees have been deposited in Zenodo at https://zenodo.org/records/20264616 (accessed on 28 May 2026, doi: 10.5281/zenodo.20264615).

## Supplementary Materials

The following supporting information can be downloaded at: https://www.mdpi.com/article/10.3390/jof12060390/s1, Figure S1: Colony morphology of some of the *Fusarium oxysporum* isolates used in this study following 10 days of growth on half-strength potato dextrose agar; Figure S2: A Maximum Likelihood tree (fitted model = TN+F+I+G4) reconstructed using concatenated sequences of translation elongation factor 1 alpha (TEF1) and the second largest subunit of RNA polymerase II (RPB2); Figure S3: A Maximum Likelihood phylogenetic placement (fitted model = GTR+G4) of *Fusarium oxysporum* f. sp. *zingiberi SIX7* genes within the *Fusarium oxysporum* species complex; Figure S4: A Maximum Likelihood phylogenetic placement (fitted model = K2P+I) of *Fusarium oxysporum* f. sp. *zingiberi SIX9* genes within the *Fusarium oxysporum* species complex; Figure S5: A Maximum Likelihood phylogenetic placement (fitted model = TN+I) of *Fusarium oxysporum* f. sp. *zingiberi SIX10* genes within the *Fusarium oxysporum* species complex; Figure S6: A Maximum Likelihood phylogenetic placement (fitted model = HKY+I) of *Fusarium oxysporum* f. sp. *zingiberi SIX12* genes within the *Fusarium oxysporum* species complex. Table S1: A total of 52 *Fusarium oxysporum* isolates from ginger plants examined in this study; Table S2: Primers used in this study; Table S3: SIX gene profiles of the isolates characterised in this study using the universal SIX gene primers [19]; Table S4: BUSCO Assessment on the Fusarium genome assemblies; Table S5: Overall statistics of orthogroup gene clusters identified using OrthoVenn3.
