# Supplementary material for: Genetic and Pathogenic Differentiation of Fusarium oxysporum Isolates from Ginger
Source: J Fungi (Basel). 2026 May 29;12(6):390. doi: 10.3390/jof12060390 (PMC13302738; doi:10.3390/jof12060390)
Supplement: Supplementary file 1 [file jof-12-00390-s001.zip › jof-4284481-supplementary.pdf]

Supplemental Materials

Genetic and pathogenic differentiation of *Fusarium oxysporum* isolates from ginger

Andrea Matthews [1], Duy P. Le [1, 2], Sharon Hamill [3], Jirah Villajuan [1], Donald M. Gardiner [4], Elizabeth A. B. Aitken [1], and Andrew Chen [1,*]

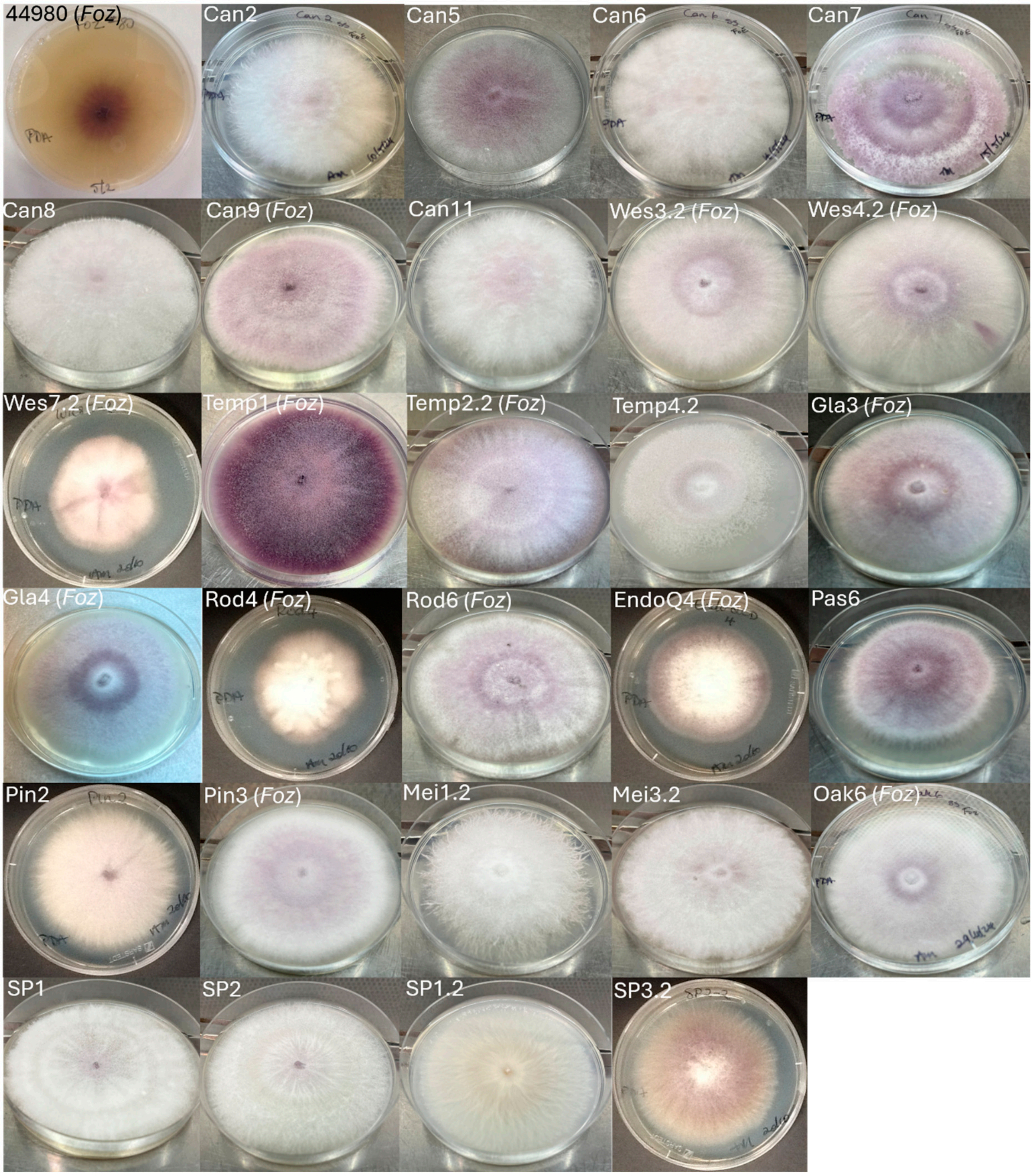

Figure S1. Colony morphology of some of the *Fusarium oxysporum* isolates used in this study following 10 days of growth on half-strength potato dextrose agar. Isolates that are pathogenic on ginger are annotated with *Foz* (*Fusarium oxysporum* f. sp. *zingiberi*).

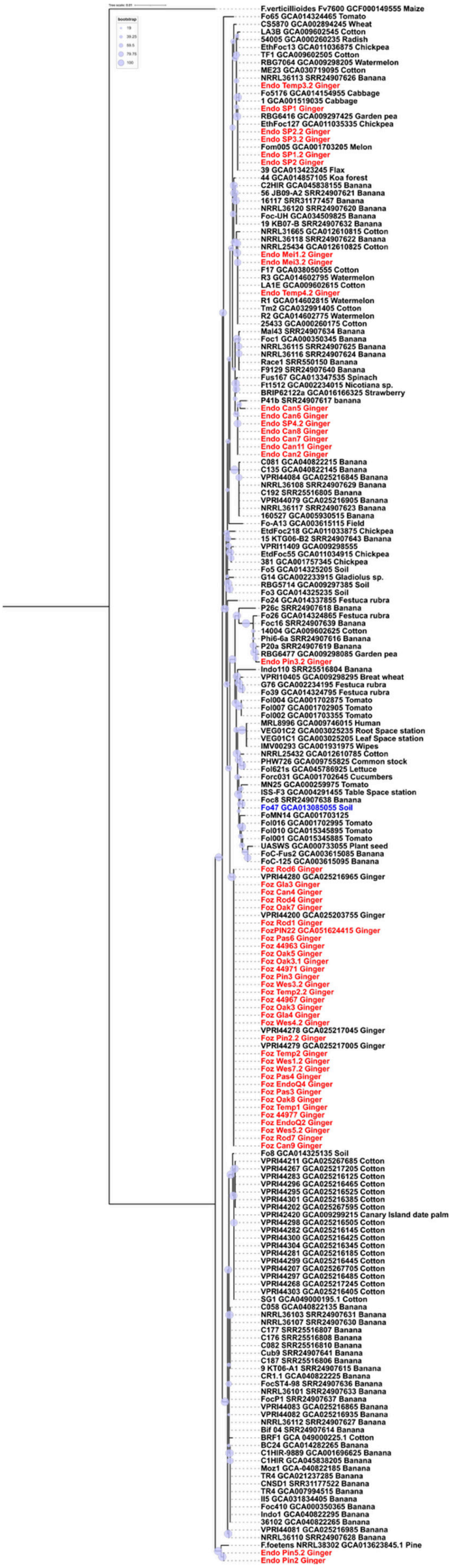
Tree scale: 0.01
bootstrap
F.verticillioides Fv7600 GCF000149555 Maize
Fo65 GCA014324465 Tomato
CS5870 GCA002894245 Wheat
LA3B GCA009602545 Cotton
54005 GCA000260235 Radish
EthFoc13 GCA011036875 Chickpea
TF1 GCA009602505 Cotton
RBG7064 GCA009298205 Watermelon
ME23 GCA030719095 Cotton
NRRL36113 SRR24907626 Banana
Endo Temp3.2 Ginger
Fo5176 GCA014154955 Cabbage
1 GCA001519035 Cabbage
Endo SP1 Ginger
RBG6416 GCA009297425 Garden pea
EthFoc127 GCA011035335 Chickpea
Endo SP2.2 Ginger
Endo SP3.2 Ginger
Fom005 GCA001703205 Melon
Endo SP1.2 Ginger
Endo SP2 Ginger
39 GCA013423245 Flax
44 GCA014857105 Koa forest
C2HIR GCA045838155 Banana
56 JB09-A2 SRR24907621 Banana
16117 SRR31177457 Banana
NRRL36120 SRR24907620 Banana
Foc-UH GCA034509825 Banana
19 KB07-B SRR24907632 Banana
NRRL31665 GCA012610815 Cotton
NRRL36118 SRR24907622 Banana
NRRL25434 GCA012610825 Cotton
Endo Mei1.2 Ginger
Endo Mei3.2 Ginger
F17 GCA038050555 Cotton
R3 GCA014602795 Watermelon
LA1E GCA009602615 Cotton
Endo Temp4.2 Ginger
R1 GCA014602815 Watermelon
Tm2 GCA032991405 Cotton
R2 GCA014602775 Watermelon
25433 GCA000260175 Cotton
Mal43 SRR24907634 Banana
Foc1 GCA000350345 Banana
NRRL36115 SRR24907625 Banana
NRRL36116 SRR24907624 Banana
Race1 SRR550150 Banana
F9129 SRR24907640 Banana
Fus167 GCA013347535 Spinach
Ft1512 GCA002234015 Nicotiana sp.
BRIP62122a GCA016166325 Strawberry
P41b SRR24907617 banana
Endo Can5 Ginger
Endo Can6 Ginger
Endo SP4.2 Ginger
Endo Can8 Ginger
Endo Can7 Ginger
Endo Can11 Ginger
Endo Can2 Ginger
C081 GCA040822215 Banana
C135 GCA040822145 Banana
VPRI44084 GCA025216845 Banana
NRRL36108 SRR24907629 Banana
C192 SRR25516805 Banana
VPRI44079 GCA025216905 Banana
NRRL36117 SRR24907623 Banana
160527 GCA005930515 Banana
Fo-A13 GCA003615115 Field
EtdFoc218 GCA011033875 Chickpea
15 KTG06-B2 SRR24907643 Banana
VPRI11409 GCA009298555
EtdFoc55 GCA011034915 Chickpea
381 GCA001757345 Chickpea
Fo5 GCA014325205 Soil
G14 GCA002233915 Gladiolus sp.
RBG5714 GCA009297385 Soil
Fo3 GCA014325235 Soil
Fo24 GCA014337855 Festuca rubra
P26c SRR24907618 Banana
Fo26 GCA014324865 Festuca rubra
Foc16 SRR24907639 Banana
14004 GCA009602625 Cotton
Phi6-6a SRR24907616 Banana
P20a SRR24907619 Banana
RBG6477 GCA009298085 Garden pea
Endo Pin3.2 Ginger
Indo110 SRR25516804 Banana
VPRI10405 GCA009298295 Breat wheat
G76 GCA002234195 Festuca rubra
Fo39 GCA014324795 Festuca rubra
Fol004 GCA001702875 Tomato
Fol007 GCA001702905 Tomato
Fol002 GCA001703355 Tomato
MRL8996 GCA009746015 Human
VEG01C2 GCA003025235 Root Space station
VEG01C1 GCA003025205 Leaf Space station
IMV00293 GCA001931975 Wipes
NRRL25432 GCA012610785 Cotton
PHW726 GCA009755825 Common stock
Fol621s GCA045786925 Lettuce
Forc031 GCA001702645 Cucumbers
MN25 GCA000259975 Tomato
ISS-F3 GCA004291455 Table Space station
Foc8 SRR24907638 Banana
Fo47 GCA013085055 Soil
FoMN14 GCA001703125
Fol016 GCA001702995 Tomato
Fol010 GCA015345895 Tomato
Fol001 GCA015345885 Tomato
UASWS GCA000733055 Plant seed
FoC-Fus2 GCA003615085 Banana
FoC-125 GCA003615095 Banana
Foz Rod6 Ginger
VPRI44280 GCA025216965 Ginger
Foz Gla3 Ginger
Foz Can4 Ginger
Foz Rod4 Ginger
Foz Oak7 Ginger
VPRI44200 GCA025203755 Ginger
Foz Rod1 Ginger
FozPIN22 GCA051624415 Ginger
Foz Pas6 Ginger
Foz 44963 Ginger
Foz Oak5 Ginger
Foz Oak3.1 Ginger
Foz 44971 Ginger
Foz Pin3 Ginger
Foz Wes3.2 Ginger
Foz Temp2.2 Ginger
Foz 44967 Ginger
Foz Oak3 Ginger
Foz Gla4 Ginger
Foz Wes4.2 Ginger
VPRI44278 GCA025217045 Ginger
Foz Pin2.2 Ginger
VPRI44279 GCA025217005 Ginger
Foz Temp2 Ginger
Foz Wes1.2 Ginger
Foz Wes7.2 Ginger
Foz Pas4 Ginger
Foz EndoQ4 Ginger
Foz Pas3 Ginger
Foz Oak8 Ginger
Foz Temp1 Ginger
Foz 44977 Ginger
Foz EndoQ2 Ginger
Foz Wes5.2 Ginger
Foz Rod7 Ginger
Foz Can9 Ginger
Fo8 GCA014325135 Soil
VPRI44211 GCA025267685 Cotton
VPRI44267 GCA025217205 Cotton
VPRI44283 GCA025216125 Cotton
VPRI44296 GCA025216465 Cotton
VPRI44295 GCA025216525 Cotton
VPRI44301 GCA025216385 Cotton
VPRI44202 GCA025267595 Cotton
VPRI42420 GCA009299215 Canary Island date palm
VPRI44298 GCA025216505 Cotton
VPRI44282 GCA025216145 Cotton
VPRI44300 GCA025216425 Cotton
VPRI44304 GCA025216345 Cotton
VPRI44281 GCA025216185 Cotton
VPRI44299 GCA025216445 Cotton
VPRI44207 GCA025267705 Cotton
VPRI44297 GCA025216485 Cotton
VPRI44268 GCA025217245 Cotton
VPRI44303 GCA025216405 Cotton
SG1 GCA049000195.1 Cotton
C058 GCA040822135 Banana
NRRL36103 SRR24907631 Banana
NRRL36107 SRR24907630 Banana
C177 SRR25516807 Banana
C176 SRR25516808 Banana
C082 SRR25516810 Banana
Cub9 SRR24907641 Banana
C187 SRR25516806 Banana
9 KT06-A1 SRR24907615 Banana
CR1.1 GCA040822225 Banana
FocST4-98 SRR24907636 Banana
NRRL36101 SRR24907633 Banana
FocP1 SRR24907637 Banana
VPRI44083 GCA025216865 Banana
VPRI44082 GCA025216935 Banana
NRRL36112 SRR24907627 Banana
Bif 04 SRR24907614 Banana
BRF1 GCA 049000225.1 Cotton
BC24 GCA014282265 Banana
C1HIR-9889 GCA001696625 Banana
C1HIR GCA045838205 Banana
Moz1 GCA-040822185 Banana
TR4 GCA021237285 Banana
CNSD1 SRR31177522 Banana
TR4 GCA007994515 Banana
II5 GCA031834405 Banana
Foc410 GCA000350365 Banana
Indo1 GCA040822295 Banana
36102 GCA040822265 Banana
VPRI44081 GCA025216985 Banana
NRRL36110 SRR24907628 Banana
F.foetens NRRL38302 GCA013623845.1 Pine
Endo Pin5.2 Ginger
Endo Pin2 Ginger

Figure S2. A Maximum Likelihood tree (fitted model = TN+F+I+G4) reconstructed using concatenated sequences of translation elongation factor 1 alpha (TEF1) and the second largest subunit of RNA polymerase II (RPB2). The endophytic and pathogenic *Fusarium oxysporum* isolates from ginger analysed in this study are highlighted in red. The *F. oxysporum* strain Fo47 with known biocontrol properties is highlighted in blue. The bar denotes a scale range of 0.01. Circles indicate bootstrap values expressed as a percentage at each node. *Fusarium verticillioides* (Fv7600) was used as an outgroup to anchor the phylogenetic tree.

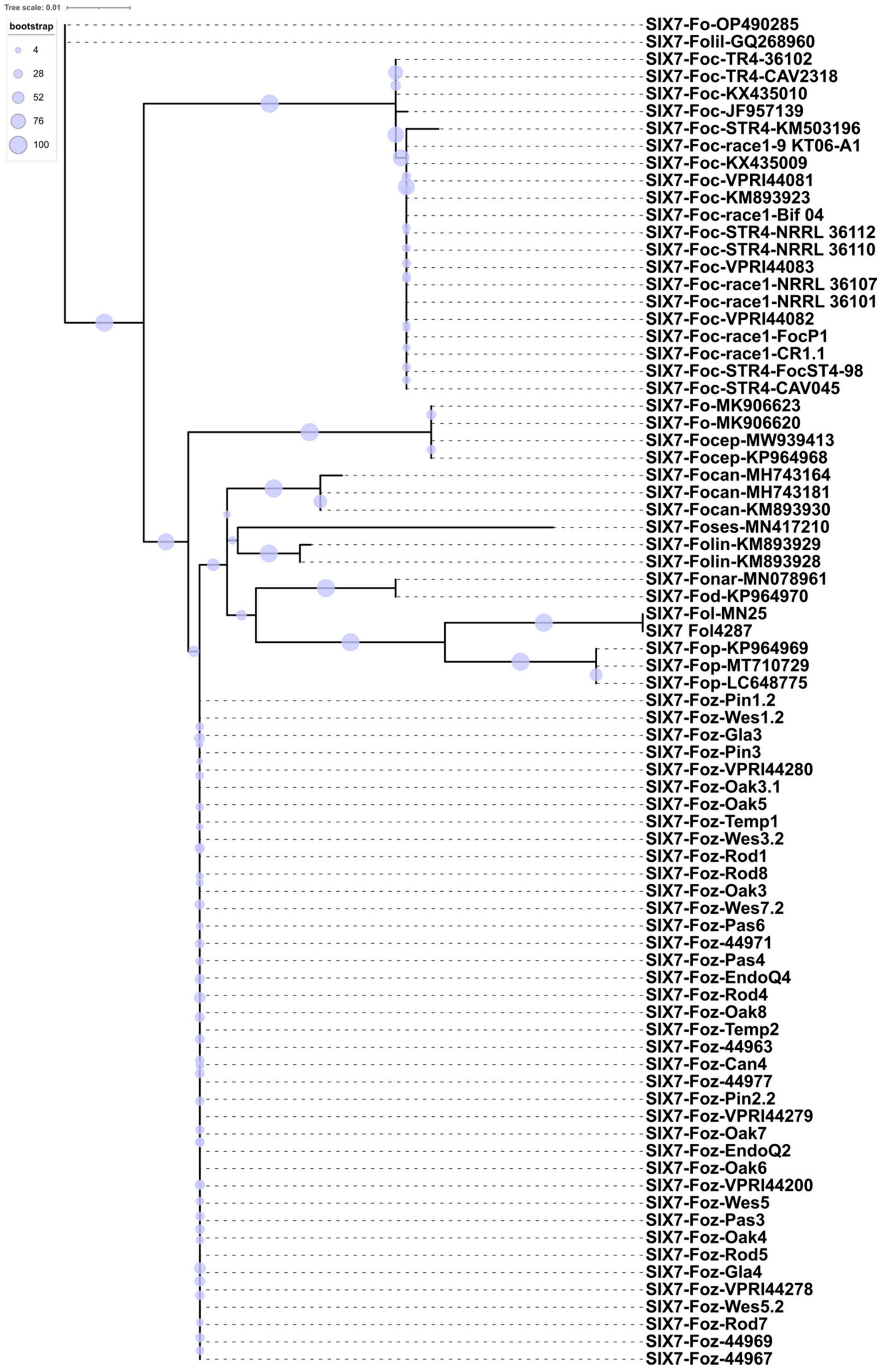
Tree scale: 0.01
bootstrap
4
28
52
76
100
SIX7-Fo-OP490285
SIX7-Folil-GQ268960
SIX7-Foc-TR4-36102
SIX7-Foc-TR4-CAV2318
SIX7-Foc-KX435010
SIX7-Foc-JF957139
SIX7-Foc-STR4-KM503196
SIX7-Foc-race1-9 KT06-A1
SIX7-Foc-KX435009
SIX7-Foc-VPRI44081
SIX7-Foc-KM893923
SIX7-Foc-race1-Bif 04
SIX7-Foc-STR4-NRRL 36112
SIX7-Foc-STR4-NRRL 36110
SIX7-Foc-VPRI44083
SIX7-Foc-race1-NRRL 36107
SIX7-Foc-race1-NRRL 36101
SIX7-Foc-VPRI44082
SIX7-Foc-race1-FocP1
SIX7-Foc-race1-CR1.1
SIX7-Foc-STR4-FocST4-98
SIX7-Foc-STR4-CAV045
SIX7-Fo-MK906623
SIX7-Fo-MK906620
SIX7-Focep-MW939413
SIX7-Focep-KP964968
SIX7-Focan-MH743164
SIX7-Focan-MH743181
SIX7-Focan-KM893930
SIX7-Foses-MN417210
SIX7-Folin-KM893929
SIX7-Folin-KM893928
SIX7-Fonar-MN078961
SIX7-Fod-KP964970
SIX7-Fol-MN25
SIX7 Fol4287
SIX7-Fop-KP964969
SIX7-Fop-MT710729
SIX7-Fop-LC648775
SIX7-Foz-Pin1.2
SIX7-Foz-Wes1.2
SIX7-Foz-Gla3
SIX7-Foz-Pin3
SIX7-Foz-VPRI44280
SIX7-Foz-Oak3.1
SIX7-Foz-Oak5
SIX7-Foz-Temp1
SIX7-Foz-Wes3.2
SIX7-Foz-Rod1
SIX7-Foz-Rod8
SIX7-Foz-Oak3
SIX7-Foz-Wes7.2
SIX7-Foz-Pas6
SIX7-Foz-44971
SIX7-Foz-Pas4
SIX7-Foz-EndoQ4
SIX7-Foz-Rod4
SIX7-Foz-Oak8
SIX7-Foz-Temp2
SIX7-Foz-44963
SIX7-Foz-Can4
SIX7-Foz-44977
SIX7-Foz-Pin2.2
SIX7-Foz-VPRI44279
SIX7-Foz-Oak7
SIX7-Foz-EndoQ2
SIX7-Foz-Oak6
SIX7-Foz-VPRI44200
SIX7-Foz-Wes5
SIX7-Foz-Pas3
SIX7-Foz-Oak4
SIX7-Foz-Rod5
SIX7-Foz-Gla4
SIX7-Foz-VPRI44278
SIX7-Foz-Wes5.2
SIX7-Foz-Rod7
SIX7-Foz-44969
SIX7-Foz-44967

Figure S3. A Maximum Likelihood phylogenetic placement (fitted model = GTR+G4) of *Fusarium oxysporum* f. sp. *zingiberi SIX7* genes within the *Fusarium oxysporum* species complex. The scale bar indicates substitutions per site. Bootstrap values expressed as a percentage are shown at each node. Trees are shown as unrooted. Abbreviations included *F. oxysporum* f. sp. *cubense* (*Foc*), *Fusarium oxysporum* f. sp. *niveum* (*Fon*), *F. oxysporum* f. sp. *sesami* (*Foses*), *F. oxysporum* f. sp. *cepae* (*Focep*), *F. oxysporum* f. sp. lycopersici, *F. oxysporum* f. sp. *lilii* (*Folil*), *F. oxysporum* f. sp. *lini* (*Folin*), *F. oxysporum* f. sp. *canariensis* (*Focan*), *F. oxysporum* f. sp. *dianthi* (*Fod*), *F. oxysporum* f. sp. *narcissi* (*Fonar*), *F. oxysporum* f. sp. *pisi* (*Fop*), *F. oxysporum* f. sp. *palmarum* (*Fopal*), *Fusarium oxysporum* f. sp. physali (*Fophy*), and *F. oxysporum* f. sp. *dactylifera* (*Fodac*).

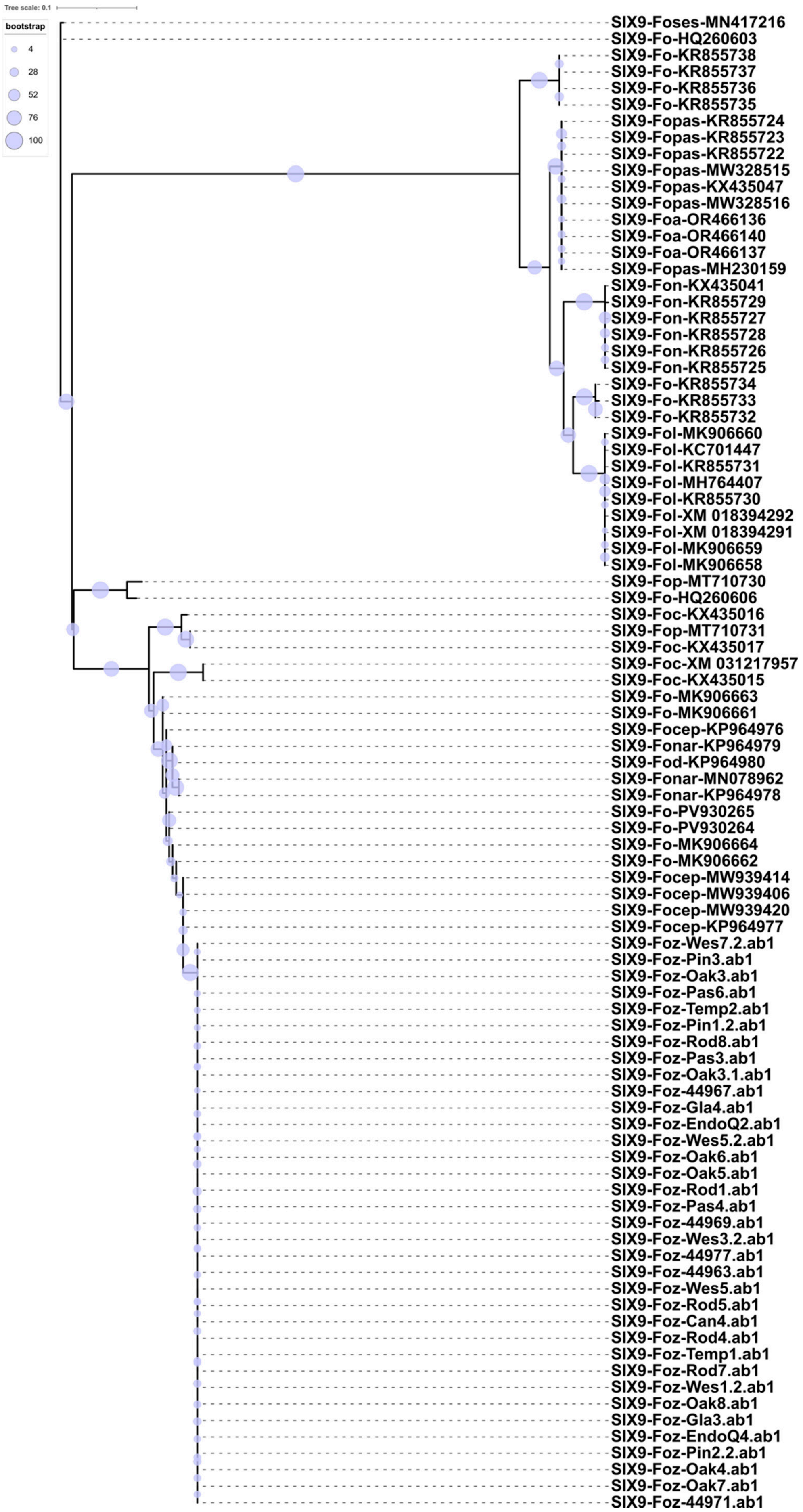
Tree scale: 0.1
bootstrap
4
28
52
76
100
SIX9-Foses-MN417216
SIX9-Fo-HQ260603
SIX9-Fo-KR855738
SIX9-Fo-KR855737
SIX9-Fo-KR855736
SIX9-Fo-KR855735
SIX9-Fopas-KR855724
SIX9-Fopas-KR855723
SIX9-Fopas-KR855722
SIX9-Fopas-MW328515
SIX9-Fopas-KX435047
SIX9-Fopas-MW328516
SIX9-Foa-OR466136
SIX9-Foa-OR466140
SIX9-Foa-OR466137
SIX9-Fopas-MH230159
SIX9-Fon-KX435041
SIX9-Fon-KR855729
SIX9-Fon-KR855727
SIX9-Fon-KR855728
SIX9-Fon-KR855726
SIX9-Fon-KR855725
SIX9-Fo-KR855734
SIX9-Fo-KR855733
SIX9-Fo-KR855732
SIX9-Fol-MK906660
SIX9-Fol-KC701447
SIX9-Fol-KR855731
SIX9-Fol-MH764407
SIX9-Fol-KR855730
SIX9-Fol-XM 018394292
SIX9-Fol-XM 018394291
SIX9-Fol-MK906659
SIX9-Fol-MK906658
SIX9-Fop-MT710730
SIX9-Fo-HQ260606
SIX9-Foc-KX435016
SIX9-Fop-MT710731
SIX9-Foc-KX435017
SIX9-Foc-XM 031217957
SIX9-Foc-KX435015
SIX9-Fo-MK906663
SIX9-Fo-MK906661
SIX9-Focep-KP964976
SIX9-Fonar-KP964979
SIX9-Fod-KP964980
SIX9-Fonar-MN078962
SIX9-Fonar-KP964978
SIX9-Fo-PV930265
SIX9-Fo-PV930264
SIX9-Fo-MK906664
SIX9-Fo-MK906662
SIX9-Focep-MW939414
SIX9-Focep-MW939406
SIX9-Focep-MW939420
SIX9-Focep-KP964977
SIX9-Foz-Wes7.2.ab1
SIX9-Foz-Pin3.ab1
SIX9-Foz-Oak3.ab1
SIX9-Foz-Pas6.ab1
SIX9-Foz-Temp2.ab1
SIX9-Foz-Pin1.2.ab1
SIX9-Foz-Rod8.ab1
SIX9-Foz-Pas3.ab1
SIX9-Foz-Oak3.1.ab1
SIX9-Foz-44967.ab1
SIX9-Foz-Gla4.ab1
SIX9-Foz-EndoQ2.ab1
SIX9-Foz-Wes5.2.ab1
SIX9-Foz-Oak6.ab1
SIX9-Foz-Oak5.ab1
SIX9-Foz-Rod1.ab1
SIX9-Foz-Pas4.ab1
SIX9-Foz-44969.ab1
SIX9-Foz-Wes3.2.ab1
SIX9-Foz-44977.ab1
SIX9-Foz-44963.ab1
SIX9-Foz-Wes5.ab1
SIX9-Foz-Rod5.ab1
SIX9-Foz-Can4.ab1
SIX9-Foz-Rod4.ab1
SIX9-Foz-Temp1.ab1
SIX9-Foz-Rod7.ab1
SIX9-Foz-Wes1.2.ab1
SIX9-Foz-Oak8.ab1
SIX9-Foz-Gla3.ab1
SIX9-Foz-EndoQ4.ab1
SIX9-Foz-Pin2.2.ab1
SIX9-Foz-Oak4.ab1
SIX9-Foz-Oak7.ab1
SIX9-Foz-44971.ab1

Figure S4. A Maximum Likelihood phylogenetic placement (fitted model = K2P+I) of *Fusarium oxysporum* f. sp. *zingiberi SIX9* genes within the *Fusarium oxysporum* species complex. The scale bar indicates substitutions per site. Bootstrap values expressed as a percentage are shown at each node. Trees are shown as unrooted. Abbreviations included *F. oxysporum* f. sp. *cubense* (*Foc*), *Fusarium oxysporum* f. sp. *niveum* (*Fon*), *F. oxysporum* f. sp. *sesami* (*Foses*), *F. oxysporum* f. sp. *cepae* (*Focep*), *F. oxysporum* f. sp. lycopersici, *F. oxysporum* f. sp. *lilii* (*Folil*), *F. oxysporum* f. sp. *lini* (*Folin*), *F. oxysporum* f. sp. *canariensis* (*Focan*), *F. oxysporum* f. sp. *dianthi* (*Fod*), *F. oxysporum* f. sp. *narcissi* (*Fonar*), *F. oxysporum* f. sp. *pisi* (*Fop*), *F. oxysporum* f. sp. *palmarum* (*Fopal*), *Fusarium oxysporum* f. sp. physali (*Fophy*), and *F. oxysporum* f. sp. *dactylifera* (*Fodac*).

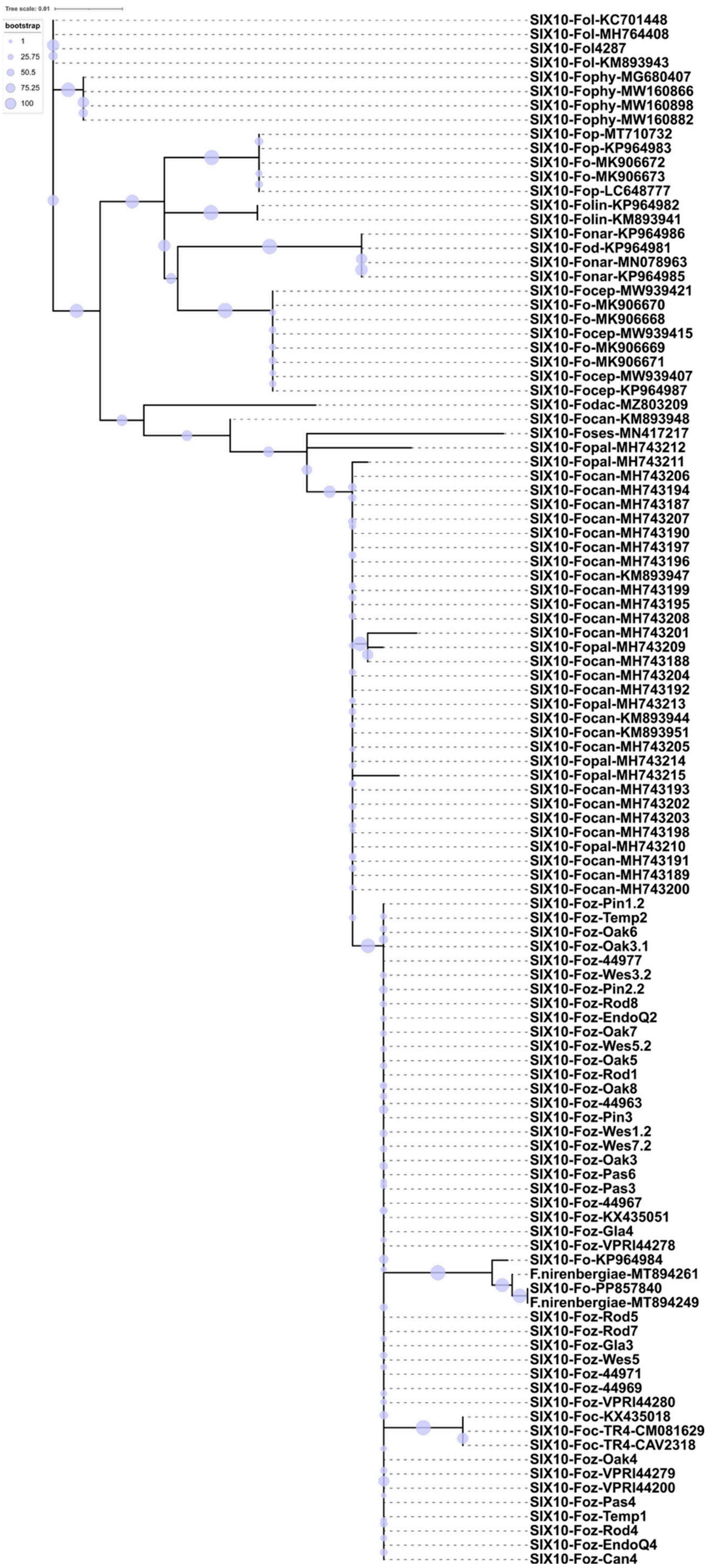
Tree scale: 0.01
bootstrap
1
25.75
50.5
75.25
100
SIX10-Fol-KC701448
SIX10-Fol-MH764408
SIX10-Fol4287
SIX10-Fol-KM893943
SIX10-Fophy-MG680407
SIX10-Fophy-MW160866
SIX10-Fophy-MW160898
SIX10-Fophy-MW160882
SIX10-Fop-MT710732
SIX10-Fop-KP964983
SIX10-Fo-MK906672
SIX10-Fo-MK906673
SIX10-Fop-LC648777
SIX10-Folin-KP964982
SIX10-Folin-KM893941
SIX10-Fonar-KP964986
SIX10-Fod-KP964981
SIX10-Fonar-MN078963
SIX10-Fonar-KP964985
SIX10-Focep-MW939421
SIX10-Fo-MK906670
SIX10-Fo-MK906668
SIX10-Focep-MW939415
SIX10-Fo-MK906669
SIX10-Fo-MK906671
SIX10-Focep-MW939407
SIX10-Focep-KP964987
SIX10-Fodac-MZ803209
SIX10-Focan-KM893948
SIX10-Foses-MN417217
SIX10-Fopal-MH743212
SIX10-Fopal-MH743211
SIX10-Focan-MH743206
SIX10-Focan-MH743194
SIX10-Focan-MH743187
SIX10-Focan-MH743207
SIX10-Focan-MH743190
SIX10-Focan-MH743197
SIX10-Focan-MH743196
SIX10-Focan-KM893947
SIX10-Focan-MH743199
SIX10-Focan-MH743195
SIX10-Focan-MH743208
SIX10-Focan-MH743201
SIX10-Fopal-MH743209
SIX10-Focan-MH743188
SIX10-Focan-MH743204
SIX10-Focan-MH743192
SIX10-Fopal-MH743213
SIX10-Focan-KM893944
SIX10-Focan-KM893951
SIX10-Focan-MH743205
SIX10-Fopal-MH743214
SIX10-Fopal-MH743215
SIX10-Focan-MH743193
SIX10-Focan-MH743202
SIX10-Focan-MH743203
SIX10-Focan-MH743198
SIX10-Fopal-MH743210
SIX10-Focan-MH743191
SIX10-Focan-MH743189
SIX10-Focan-MH743200
SIX10-Foz-Pin1.2
SIX10-Foz-Temp2
SIX10-Foz-Oak6
SIX10-Foz-Oak3.1
SIX10-Foz-44977
SIX10-Foz-Wes3.2
SIX10-Foz-Pin2.2
SIX10-Foz-Rod8
SIX10-Foz-EndoQ2
SIX10-Foz-Oak7
SIX10-Foz-Wes5.2
SIX10-Foz-Oak5
SIX10-Foz-Rod1
SIX10-Foz-Oak8
SIX10-Foz-44963
SIX10-Foz-Pin3
SIX10-Foz-Wes1.2
SIX10-Foz-Wes7.2
SIX10-Foz-Oak3
SIX10-Foz-Pas6
SIX10-Foz-Pas3
SIX10-Foz-44967
SIX10-Foz-KX435051
SIX10-Foz-Gla4
SIX10-Foz-VPRI44278
SIX10-Fo-KP964984
F.nirenbergiae-MT894261
SIX10-Fo-PP857840
F.nirenbergiae-MT894249
SIX10-Foz-Rod5
SIX10-Foz-Rod7
SIX10-Foz-Gla3
SIX10-Foz-Wes5
SIX10-Foz-44971
SIX10-Foz-44969
SIX10-Foz-VPRI44280
SIX10-Foc-KX435018
SIX10-Foc-TR4-CM081629
SIX10-Foc-TR4-CAV2318
SIX10-Foz-Oak4
SIX10-Foz-VPRI44279
SIX10-Foz-VPRI44200
SIX10-Foz-Pas4
SIX10-Foz-Temp1
SIX10-Foz-Rod4
SIX10-Foz-EndoQ4
SIX10-Foz-Can4

Figure S5. A Maximum Likelihood phylogenetic placement (fitted model = TN+I) of *Fusarium oxysporum* f. sp. *zingiberi SIX10* genes within the *Fusarium oxysporum* species complex. The scale bar indicates substitutions per site. Bootstrap values expressed as a percentage are shown at each node. Trees are shown as unrooted. Abbreviations included *F. oxysporum* f. sp. *cubense* (*Foc*), *Fusarium oxysporum* f. sp. *niveum* (*Fon*), *F. oxysporum* f. sp. *sesami* (*Foses*), *F. oxysporum* f. sp. *cepae* (*Focep*), *F. oxysporum* f. sp. lycopersici, *F. oxysporum* f. sp. *lilii* (*Folil*), *F. oxysporum* f. sp. *lini* (*Folin*), *F. oxysporum* f. sp. *canariensis* (*Focan*), *F. oxysporum* f. sp. *dianthi* (*Fod*), *F. oxysporum* f. sp. *narcissi* (*Fonar*), *F. oxysporum* f. sp. *pisi* (*Fop*), *F. oxysporum* f. sp. *palmarum* (*Fopal*), *Fusarium oxysporum* f. sp. physali (*Fophy*), and *F. oxysporum* f. sp. *dactylifera* (*Fodac*).

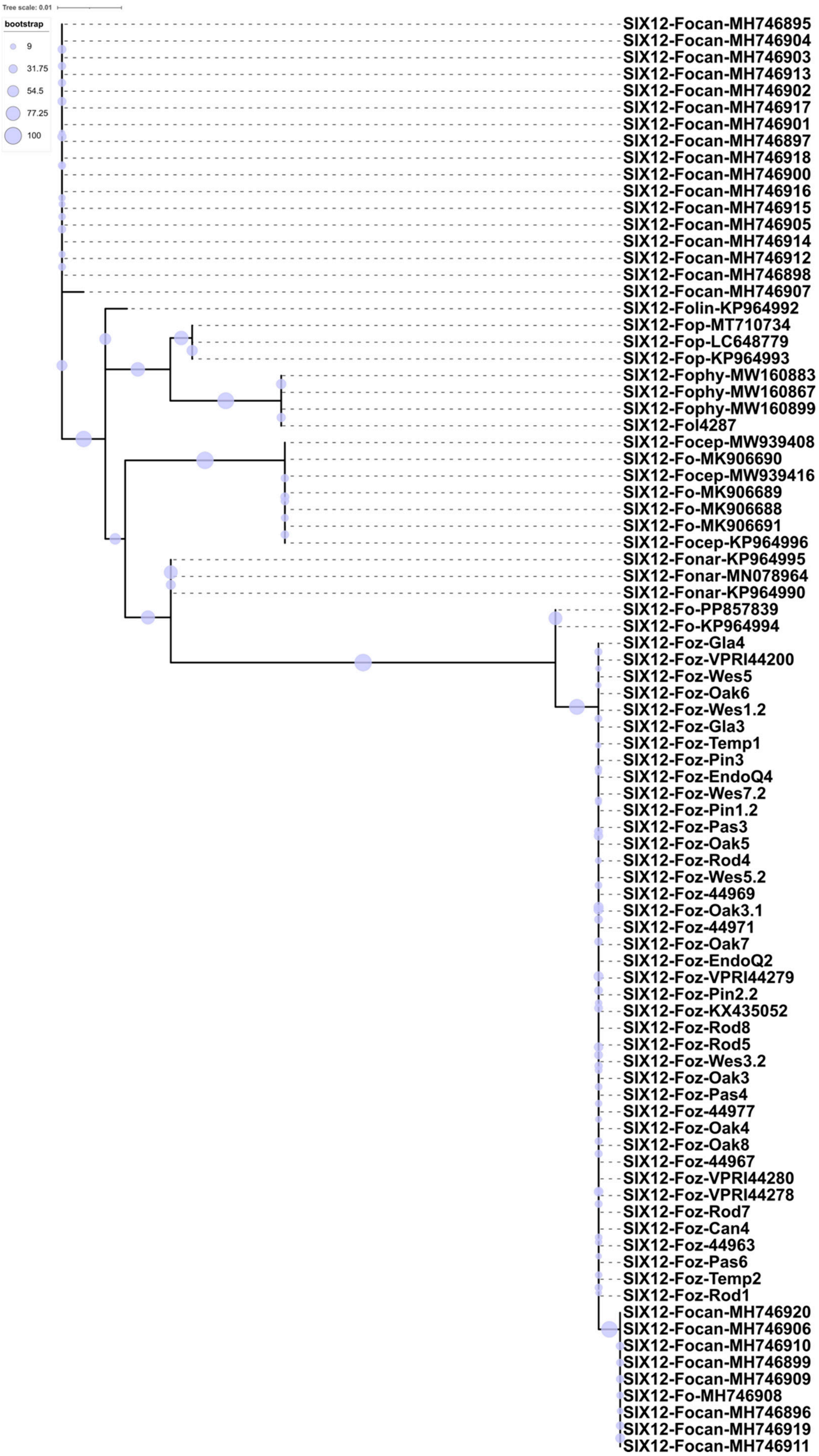
Tree scale: 0.01
bootstrap
9
31.75
54.5
77.25
100
SIX12-Focan-MH746895
SIX12-Focan-MH746904
SIX12-Focan-MH746903
SIX12-Focan-MH746913
SIX12-Focan-MH746902
SIX12-Focan-MH746917
SIX12-Focan-MH746901
SIX12-Focan-MH746897
SIX12-Focan-MH746918
SIX12-Focan-MH746900
SIX12-Focan-MH746916
SIX12-Focan-MH746915
SIX12-Focan-MH746905
SIX12-Focan-MH746914
SIX12-Focan-MH746912
SIX12-Focan-MH746898
SIX12-Focan-MH746907
SIX12-Folin-KP964992
SIX12-Fop-MT710734
SIX12-Fop-LC648779
SIX12-Fop-KP964993
SIX12-Fophy-MW160883
SIX12-Fophy-MW160867
SIX12-Fophy-MW160899
SIX12-Fol4287
SIX12-Focep-MW939408
SIX12-Fo-MK906690
SIX12-Focep-MW939416
SIX12-Fo-MK906689
SIX12-Fo-MK906688
SIX12-Fo-MK906691
SIX12-Focep-KP964996
SIX12-Fonar-KP964995
SIX12-Fonar-MN078964
SIX12-Fonar-KP964990
SIX12-Fo-PP857839
SIX12-Fo-KP964994
SIX12-Foz-Gla4
SIX12-Foz-VPRI44200
SIX12-Foz-Wes5
SIX12-Foz-Oak6
SIX12-Foz-Wes1.2
SIX12-Foz-Gla3
SIX12-Foz-Temp1
SIX12-Foz-Pin3
SIX12-Foz-EndoQ4
SIX12-Foz-Wes7.2
SIX12-Foz-Pin1.2
SIX12-Foz-Pas3
SIX12-Foz-Oak5
SIX12-Foz-Rod4
SIX12-Foz-Wes5.2
SIX12-Foz-44969
SIX12-Foz-Oak3.1
SIX12-Foz-44971
SIX12-Foz-Oak7
SIX12-Foz-EndoQ2
SIX12-Foz-VPRI44279
SIX12-Foz-Pin2.2
SIX12-Foz-KX435052
SIX12-Foz-Rod8
SIX12-Foz-Rod5
SIX12-Foz-Wes3.2
SIX12-Foz-Oak3
SIX12-Foz-Pas4
SIX12-Foz-44977
SIX12-Foz-Oak4
SIX12-Foz-Oak8
SIX12-Foz-44967
SIX12-Foz-VPRI44280
SIX12-Foz-VPRI44278
SIX12-Foz-Rod7
SIX12-Foz-Can4
SIX12-Foz-44963
SIX12-Foz-Pas6
SIX12-Foz-Temp2
SIX12-Foz-Rod1
SIX12-Focan-MH746920
SIX12-Focan-MH746906
SIX12-Focan-MH746910
SIX12-Focan-MH746899
SIX12-Focan-MH746909
SIX12-Fo-MH746908
SIX12-Focan-MH746896
SIX12-Focan-MH746919
SIX12-Focan-MH746911

Figure S6. A Maximum Likelihood phylogenetic placement (fitted model = HKY+I) of *Fusarium oxysporum* f. sp. *zingiberi SIX12* genes within the *Fusarium oxysporum* species complex. The scale bar indicates substitutions per site. Bootstrap values expressed as a percentage are shown at each node. Trees are shown as unrooted. Abbreviations included *F. oxysporum* f. sp. *cubense* (*Foc*), *Fusarium oxysporum* f. sp. *niveum* (*Fon*), *F. oxysporum* f. sp. *sesami* (*Foses*), *F. oxysporum* f. sp. *cepae* (*Focep*), *F. oxysporum* f. sp. lycopersici, *F. oxysporum* f. sp. *lilii* (*Folil*), *F. oxysporum* f. sp. *lini* (*Folin*), *F. oxysporum* f. sp. *canariensis* (*Focan*), *F. oxysporum* f. sp. *dianthi* (*Fod*), *F. oxysporum* f. sp. *narcissi* (*Fonar*), *F. oxysporum* f. sp. *pisi* (*Fop*), *F. oxysporum* f. sp. *palmarum* (*Fopal*), *Fusarium oxysporum* f. sp. physali (*Fophy*), and *F. oxysporum* f. sp. *dactylifera* (*Fodac*).

Table S1. A total of 52 *Fusarium oxysporum* isolates from ginger plants examined in this study.

**Isolate name**	**Accession number**	**Location**	**Plant host**	**Symptoms**	**GPS Coordinates**
Can2	UQ6451	Eumundi	Ginger after sugarcane	Fusarium yellows	-26.4, 152.9
Can4	UQ6453	Eumundi	Ginger after sugarcane	Fusarium yellows	-26.4, 152.9
Can5	UQ6454	Eumundi	Ginger after sugarcane	Fusarium yellows	-26.4, 152.9
Can6	UQ6455	Eumundi	Ginger after sugarcane	Fusarium yellows	-26.4, 152.9
Can7	UQ6456	Eumundi	Ginger after sugarcane	Fusarium yellows	-26.4, 152.9
Can8	UQ6457	Eumundi	Ginger after sugarcane	Fusarium yellows	-26.4, 152.9
Can9	UQ6458	Eumundi	Ginger after sugarcane	Fusarium yellows	-26.4, 152.9
Can11	UQ6460	Eumundi	Ginger after sugarcane	Fusarium yellows	-26.4, 152.9
Wes1.2	UQ6429	Yandina Creek	Ginger after 2 years fallow	Fusarium yellows	-26.5, 153.0
Wes3.2	UQ6431	Yandina Creek	Ginger after 2 years fallow	Fusarium yellows	-26.5, 153.0
Wes4.2	UQ6432	Yandina Creek	Ginger after 2 years fallow	Fusarium yellows	-26.5, 153.0
Wes5	UQ6816	Unknown	Unknown	Unknown	Unknown
Wes5.2	UQ6433	Yandina Creek	Ginger after 2 years fallow	Fusarium yellows	-26.5, 153.0
Wes7.2	UQ6435	Yandina Creek	Ginger after 2 years fallow	Fusarium yellows	-26.5, 153.0
Temp1	UQ6440	Eumundi	Ginger after 5 years fallow	Fusarium yellows	-26.4, 152.9
Temp2	UQ6441	Eumundi	Ginger after 5 years fallow	Fusarium yellows	-26.4, 152.9
Temp2.2	UQ6443	Eumundi	Ginger after 5 years fallow	Fusarium yellows	-26.4, 152.9

Isolate name	Accession number	Location	Plant host	Symptoms	GPS Coordinates
Temp3.2	UQ6444	Eumundi	Ginger after 5 years fallow	Fusarium yellows	-26.4, 152.9
Temp4.2	UQ6445	Eumundi	Ginger after 5 years fallow	Fusarium yellows	-26.4, 152.9
Gla3	UQ6876	Eumundi	Ginger after gladioli	Fusarium yellows	-26.4, 152.9
Gla4	UQ6877	Eumundi	Ginger after gladioli	Fusarium yellows	-26.4, 152.9
Rod1	UQ6469	Eumundi	Ginger after gladioli	Fusarium yellows	-26.4, 152.9
Rod4	UQ6470	Eumundi	Ginger after gladioli	Fusarium yellows	-26.4, 152.9
Rod5*	UQ6471	Eumundi	Ginger after gladioli	Fusarium yellows	-26.4, 152.9
Rod6	UQ6472	Eumundi	Ginger after gladioli	Fusarium yellows	-26.4, 152.9
Rod7	UQ6473	Eumundi	Ginger after gladioli	Fusarium yellows	-26.4, 152.9
Rod8	UQ6474	Eumundi	Ginger after gladioli	Fusarium yellows	-26.4, 152.9
EndoQ2	UQ6426	Unknown	Healthy clean Qld cv for endophytes	Healthy ginger	Unknown
EndoQ4	UQ6428	Unknown	Healthy clean Qld cv for endophytes	Healthy ginger	Unknown
Pas3	UQ6423	Unknown	Healthy looking ginger rhizomes after pasture	Fusarium yellows	Unknown
Pas4	UQ6424	Unknown	Healthy looking ginger rhizomes after pasture	Fusarium yellows	Unknown
Pas6	UQ6879	Unknown	Healthy looking ginger rhizomes after pasture	Fusarium yellows	Unknown
Pin1.2	UQ6802	Beerwah	Ginger after pineapple	Fusarium yellows	-26.8, 152.9
Pin2	UQ6448	Beerwah	Ginger after pineapple	Fusarium yellows	-26.8, 152.9
Pin2.2	UQ6803	Beerwah	Ginger after pineapple	Fusarium yellows	-26.8, 152.9
Pin3	UQ6449	Beerwah	Ginger after pineapple	Fusarium yellows	-26.8, 152.9
Pin5.2	UQ6805	Beerwah	Ginger after pineapple	Fusarium yellows	-26.8, 152.9

Isolate name	Accession number	Location	Plant host	Symptoms	GPS Coordinates
Mei1.2	UQ6437	Eumundi	Ginger after pineapple	Fusarium yellows	-26.4, 152.9
Mei3.2	UQ6439	Eumundi	Ginger after pineapple	Fusarium yellows	-26.4, 152.9
Oak3	UQ6462	Eumundi	Ginger after pineapple	Fusarium yellows	-26.4, 152.9
Oak3.1	UQ6806	Eumundi	Ginger after pineapple	Fusarium yellows	-26.4, 152.9
Oak4	UQ6463	Eumundi	Ginger after pineapple	Fusarium yellows	-26.4, 152.9
Oak5	UQ6464	Eumundi	Ginger after pineapple	Fusarium yellows	-26.4, 152.9
Oak6*	UQ6465	Eumundi	Ginger after pineapple	Fusarium yellows	-26.4, 152.9
Oak7	UQ6466	Eumundi	Ginger after pineapple	Fusarium yellows	-26.4, 152.9
Oak8	UQ6467	Eumundi	Ginger after pineapple	Fusarium yellows	-26.4, 152.9
SP1	UQ6446	Bundaberg	Ginger after sweet potato	Fusarium yellows	-24.8, 152.3
SP2	UQ6447	Bundaberg	Ginger after sweet potato	Fusarium yellows	-24.8, 152.3
SP1.2	UQ6815	Bundaberg	Ginger after sweet potato	Fusarium yellows	-24.8, 152.3
SP2.2	UQ6799	Bundaberg	Ginger after sweet potato	Fusarium yellows	-24.8, 152.3
SP3.2	UQ6800	Bundaberg	Ginger after sweet potato	Fusarium yellows	-24.8, 152.3
SP4.2	UQ6801	Bundaberg	Ginger after sweet potato	Fusarium yellows	-24.8, 152.3

* These isolates were not included in the concatenated phylogeny of TEF1 and RPB2 due to unsuccessful amplification of RPB2.

Table S2. Primers used in this study.

Gene	Name	Sequence (5' to 3')	Amplicon size (bp)	Td (°C)	Extension time	Reference
TEF1-α	EF1	ATGGGTAAGGARGACAAGAC	620-650	55 °C	1m	O'Donnell *et al.* 1998
	EF2	GGARGTACCAGTSATCATGTT				O'Donnell *et al.* 1998
RPB2	5F2	GGGGWGAYCAGAAGAAGGC	860 (5F2/7cR)	55 °C	1m 30s	O'Donnell *et al.* 2013
	7cR	CCCATRGCTTGYTTRCCCAT				Liu *et al.* 1999
	7cF	ATGGGYAARCAAGCYATGGG	917 (7cF/11aR)	53 °C	1m 30s	Liu *et al.* 1999
	11aR	GCRTGGATCTTRTCRTCSACC	1777 (5F2/11aR)	58 °C	2m 30s	Liu *et al.* 1999
SIX7	F1	CAGATTATGAAGTACCTTTACC	645	55 °C	1m	This study
	R1	CTAACTCTGCTGTGTTAAACT				This study
SIX9	F1	GCTTTTAGCAGTTGCGGCA	340	53 °C	30s	This study
	R1	CAGTTGCGGCAGTGGCTTTTG				This study

SIX10	F1	CAGTTGATTCCTCTTGTCGC	508	54 °C	1m	This study
	R1	GTAACAATTACCCAGTCTAAGTAG				This study
SIX12	F1	ATACTGCTTCAAGTACAAAC	342	52 °C	30s	This study
	R1	CAAGCTATGCCACTCCTGA				This study

Table S3. SIX gene profiles of the isolates characterised in this study using the universal SIX gene primers (Czislowski et al. 2021). *These endophytes carry single-copy *SIX9* homologs which were retrieved from the short-read genome assemblies (unpublished) of each of these isolates, thereby confirming their presence.

**Isolate Name**	**Accession Number**	***SIX 1***	***SIX 2***	***SIX 3***	***SIX 4***	***SIX 5***	***SIX 6***	***SIX 7***	***SIX 8***	***SIX9* (g1)**	***SIX9* (g2)**	***SIX 10***	***SIX 11***	***SIX 12***	***SIX 13***	***SIX 14***
44963	BRIP							+		+		+		+		
44967	BRIP							+		+		+		+		
44969	BRIP							+		+		+		+		
44971	BRIP							+		+		+		+		
44977	BRIP							+		+		+		+		
Can2	UQ6451	-	-	-	-	-	-	-	-	-	-	-	-	-	-	-
Can4	UQ6453	-	-	-	-	-	-	+	-	+	-	+	-	+	-	-
Can5	UQ6454	-	-	-	-	-	-	-	-	-	-	-	-	-	-	-
Can6	UQ6455	-	-	-	-	-	-	-	-	-	-	-	-	-	-	-
Can7	UQ6456							-		-		-		-		
Can8	UQ6457							-		-		-		-		
Can9	UQ6458							+		+		+		+		
Can11	UQ6460							-		-		-		-		
EndoQ2	UQ6426	-	-	-	-	-	-	+	-	+	-	+	-	+	-	-
EndoQ4	UQ6428	-	-	-	-	-	-	+	-	+	-	+	-	+	-	-
Gla3	UQ6876							+		+		+		+		
Gla4	UQ6877							+		+		+		+		
Mei1.2	UQ6437							-		-		-		-		
Mei3.2	UQ6439							-		-		-		-		
Oak3	UQ6462	-	-	-	-	-	-	+	-	+	-	+	-	+	-	-
Oak3.1	UQ6806	-	-	-	-	-	-	+	-	+	-	+	-	+	-	-
Oak4	UQ6463	-	-	-	-	-	-	+	-	+	-	+	-	+	-	-
Oak5	UQ6464	-	-	-	-	-	-	+	-	+	-	+	-	+	-	-
Oak6	UQ6465	-	-	-	-	-	-	+	-	+	-	+	-	+	-	-
Oak7	UQ6466	-	-	-	-	-	-	+	-	+	-	+	-	+	-	-
Oak8	UQ6467	-	-	-	-	-	-	+	-	+	-	+	-	+	-	-
Pas3	UQ6423	-	-	-	-	-	-	+	-	+	-	+	-	+	-	-

Isolate Name	Accession Number	*SIX 1*	*SIX 2*	*SIX 3*	*SIX 4*	*SIX 5*	*SIX 6*	*SIX 7*	*SIX 8*	*SIX9 (g1)*	*SIX9 (g2)*	*SIX 10*	*SIX 11*	*SIX 12*	*SIX 13*	*SIX 14*
Pas4	UQ6424	-	-	-	-	-	-	+	-	+	-	+	-	+	-	-
Pas6																
Pin1.2	UQ6802	-	-	-	-	-	-	+	-	+	-	+	-	+	-	-
Pin2	UQ6448	-	-	-	-	-	-	-	-	-	-	-	-	-	-	-
Pin2.2	UQ6803	-	-	-	-	-	-	+	-	+	-	+	-	+	-	-
Pin3	UQ6449							+		+		+		+		
Pin5.2	UQ6805	-	-	-	-	-	-	-	-	-	-	-	-	-	-	-
Rod1	UQ6469	-	-	-	-	-	-	+	-	+	-	+	-	+	-	-
Rod4	UQ6470	-	-	-	-	-	-	+	-	+	-	+	-	+	-	-
Rod5	UQ6471	-	-	-	-	-	-	+	-	+	-	+	-	+	-	-
Rod6	UQ6472							+		+		+		+		
Rod7	UQ6473	-	-	-	-	-	-	+	-	+	-	+	-	+	-	-
Rod8	UQ6474	-	-	-	-	-	-	+	-	+	-	+	-	+	-	-
SP1	UQ6446							-		+		-		-		
SP1.2*	UQ6815							-		+		-		-		
SP2*	UQ6447							-		+		-		-		
SP2.2*	UQ6799	-	-	-	-	-	-	-	-	+	-	-	-	-	-	-
SP3.2*	UQ6800	-	-	-	-	-	-	-	-	+	-	-	-	-	-	-
SP4.2*	UQ6801	-	-	-	-	-	-	-	-	+	-	-	-	-	-	-
Temp1	UQ6440	-	-	-	-	-	-	+	-	+	-	+	-	+	-	-
Temp2	UQ6441							+		+		+		+		
Temp2.2	UQ6443							+		+		+		+		
Temp3.2	UQ6798	-	-	-	-	-	-	-	-	+	-	-	-	-	-	-
Temp4.2	UQ6445							-		-		-		-		
Wes1.2	UQ6429	-	-	-	-	-	-	+	-	+	-	+	-	+	-	-
Wes3.2	UQ6431							+		+		+		+		
Wes4.2	UQ6432							+		+		+		+		
Wes5	UQ6816							+		+		+		+		
Wes5.2	UQ6433	-	-	-	-	-	-	+	-	+	-	+	-	+	-	-
Wes7.2	UQ6435	-	-	-	-	-	-	+	-	+	-	+	-	+	-	-

Table S4. BUSCO Assessment on the Fusarium genome assemblies.

Assembly	ASM14955v1	CAV2318.1	ASM5162441v1	ASM1797961v1	FoC_Fus2_v1	ASM1342324v1	ASM4583786v1	ASM14995v2
Strain	7600	CAV2318	Pin2.2	MR4003	FoC_Fus2	39	AJ275	4287
Species	*F. verticillioides*	*F. oxysporum*	*F. oxysporum*	*F. oxysporum*	*F. oxysporum*	*F. oxysporum*	*F. oxysporum*	*F. oxysporum*
Host	Culture	Banana	Ginger	Sesame	Onion	Flax	Narcissus	Tomato
NCBI accession	GCA_000149555.1	Not available	GCA_051624415.1	GCA_017979615.1	GCA_003615085.1	GCA_013423245.1	GCA_045837865.1	GCF_000149955.1
Assembly size	41885085	45183396	53509504	71179250	53425788	69459321	59641877	61471697
No. scaffold	24	522	95	96	34	26	34	88
Scaffold N50	4 Mb	264 Kb	4 Mb	3 Mb	4 Mb	5 Mb	4 Mb	4 Mb
BUSCO Complete Total	98.5% (2784)	99.0% (2798)	99.0% (2798)	98.8% (2793)	98.9% (2795)	98.9% (2796)	98.9% (2796)	96.7% (2732)
Complete and single-copy	98.1% (2771)	98.4% (2781)	98.0% (2770)	97.3% (2749)	97.9% (2767)	97.6% (2759)	97.7% (2760)	95.6% (2702)
Complete and duplicated	0.5% (13)	0.6% (17)	1.0% (28)	1.6% (44)	1.0% (28)	1.3% (37)	1.3% (36)	1.1% (30)
Fragmented	0.8% (23)	0.5% (15)	0.5% (14)	0.6% (18)	0.6% (18)	0.6% (16)	0.6% (16)	1.7% (48)
Missing	0.7% (19)	0.5% (13)	0.5% (14)	0.5% (15)	0.5% (13)	0.5% (14)	0.5% (14)	1.6% (46)
Total	2826	2826	2826	2826	2826	2826	2826	2826

Table S5. Overall statistics of orthogroup gene clusters identified using OrthoVenn3.

Result Statistics	
All clusters	20094
Single-copy clusters	6807
All proteins	155304
All singletons	5853
Percentage of singletons	3.77%
